# Overall and modality-specific exercise doses for motor skill improvement in cerebral palsy: a systematic review and Bayesian network dose-response meta-analysis

**DOI:** 10.7717/peerj.21035

**Published:** 2026-04-08

**Authors:** Qiang Xiong, Xing-liang Duan, Peng-wei He

**Affiliations:** 1 The College of Physical Education, Minnan Normal University, Zhangzhou, Fujian, China; 2 Nanfang College Guangzhou, Guangzhou, Guangdong, China

**Keywords:** Exercise, Dose, Adolescents, Cerebral palsy, Motor skills

## Abstract

**Objective:**

To examine the nonlinear dose–response of overall and modality-specific exercise interventions on motor skill improvement in children and adolescents with cerebral palsy (CP) using a Bayesian model-based network meta-analysis.

**Methods:**

Randomized controlled trials (RCTs) involving participants aged ≤18 years with cerebral palsy (CP) were retrieved from five databases (PubMed, Embase, Web of Science, Cochrane Library, SPORTDiscus; up to Aug 10, 2025). Gross motor function, assessed using the Gross Motor Function Measure (GMFM-66/88), was the main outcome. Exercise dose was standardized as metabolic equivalents (METs) × minutes per week, and model-based network meta-analysis (MBNMA) was used to estimate overall and modality-specific nonlinear effects. Study quality and evidence certainty were evaluated using the Physiotherapy Evidence Database scale (PEDro) and the Grading of Recommendations Assessment, Development and Evaluation (GRADE) framework.

**Results:**

Twenty randomized controlled trials were included. Most studies applied aerobic exercise, body control training, or resistance training. The mean PEDro score was 6.7, indicating moderate–high quality. Overall, exercise improved GMFM scores with a small-to-moderate effect (standardized mean difference (SMD) = 0.295; 95% credible interval (CrI) 0.016–0.613). The dose–response relationship showed an inverted U-shape, peaking near 560 METs × min/week, with stable gains between 330–560. By modality, body control training yielded the most consistent improvements at ~330 METs × min/week (SMD = 0.313; 95% CrI 0.014–0.666), while aerobic and resistance training showed smaller and less stable effects that declined at higher doses. Evidence certainty was moderate, with minimal publication bias.

**Conclusion:**

Exercise improved motor function in children with cerebral palsy, with optimal benefits observed at 330–560 METs × min/week. Body control training around 330 METs × min/week produced the most stable gains, whereas aerobic and resistance training declined at higher doses. These findings highlight the importance of defining effective dose ranges; larger multicenter RCTs with standardized dose reporting are needed to refine clinical guidelines.

## Introduction

Cerebral palsy (CP) is a non-progressive disorder of the central nervous system caused by brain injury during the fetal or infant stage. Its most prominent feature is impaired motor and postural development, often accompanied by sensory, cognitive, language, and emotional difficulties (Jahan et al., 2021; Rosenbaum et al., 2007; Novak et al., 2012). Epidemiological surveys report that even in developed countries with advanced medical care, the prevalence remains about four cases per 1,000 live births, with higher rates observed in developing regions (Rouabhi et al., 2023; Furtado et al., 2022). Motor impairments lead to severe difficulties in walking, postural control, and coordination, restricting social participation and reducing quality of life. Beyond individual disability, CP also exerts broader social and economic impacts, including increased healthcare demands, financial burdens on families, and barriers to social integration (Damiano & Longo, 2021; Paul et al., 2022).

Deficits in motor skills are widespread among individuals with CP, typically manifesting as poor coordination, impaired balance and postural control, and delayed development of fundamental movement patterns. The severity of motor impairment often determines independence and social participation, sometimes exerting a greater influence than associated cognitive or sensory symptoms (Plotas et al., 2024; Cabral et al., 2023). Although CP is a lifelong neurodevelopmental condition, functional performance evolves with growth and maturation, underscoring the ongoing importance of rehabilitation training in enhancing motor skills (Naaris et al., 2024). Accumulating evidence shows that exercise-based interventions can improve motor function in this population (Velasco Aguado et al., 2025). However, uncertainties remain regarding the optimal dose and the differential effects of specific modalities, highlighting the need for systematic investigation.

Most prior studies have emphasized whether exercise is effective, while insufficient attention has been paid to the critical question of how much exercise is optimal (Crispim et al., 2023). Motor skill improvement depends not merely on the presence of intervention but on the precise dose, as variations in intensity, frequency, and duration may yield markedly different outcomes. Inadequate doses may limit benefits, whereas excessive doses may induce fatigue or reduce adherence (Gebel et al., 2018; Sudati et al., 2024). Without a systematic analysis of the dose-response relationship, rehabilitation programs often rely on experience rather than evidence, leaving clinical practice without clear guidance. Determining appropriate exercise doses for children with CP can maximize improvements in motor skills and achieve optimal outcomes under limited rehabilitation resources (Ghai & Ghai, 2019; Jackman et al., 2020).

We conducted a systematic review and Bayesian dose response meta analysis to examine how total exercise dose, defined by intensity, frequency, and duration, is associated with changes in GMFM scores and whether different exercise modalities show distinct dose response patterns in children and adolescents with cerebral palsy. The GMFM, particularly its validated 66 item and 88 item versions, is the most commonly used and well validated tool for assessing gross motor function in CP and was therefore chosen as the primary outcome measure (Abe, Higashi & Himuro, 2025). In the analysis, the two versions were distinguished or standardized where necessary to ensure comparability. Establishing dose response curves is essential to allow meaningful comparison across trials and to guide evidence based rehabilitation practice. By modeling both overall trends and modality specific associations, this study aimed to identify optimal dose ranges and to provide more precise recommendations for clinical application.

## Methodology

### Literature sources and search procedure

The design and conduct of this study followed the PRISMA guidelines (Page et al., 2020) and was prospectively registered in PROSPERO (CRD420251127747) to ensure transparency and traceability. A systematic search was performed across five major databases-PubMed, Embase, Web of Science, Cochrane Library, and SPORTDiscus-covering all records up to 10 August 2025. Search strategies were tailored to each database using controlled vocabulary, free-text terms, and Boolean operators to maximize both sensitivity and precision. In addition, citation tracking of relevant systematic reviews published within the past decade was conducted to minimize the risk of missing eligible studies. To ensure that recently published studies were not missed between the initial search and manuscript revision, an updated literature search was conducted on 24 January 2026 using the same search strategy and eligibility criteria. This updated search did not identify any additional eligible studies, and therefore did not alter the study selection results. The literature search was independently conducted by Qiang Xiong and Xing-liang Duan, who also performed the initial screening and full-text assessments. All discrepancies arising during study selection, data extraction, or risk-of-bias evaluation were resolved through discussion. When consensus could not be reached, a third reviewer, Peng-wei He, acted as the adjudicator. The complete search strategies and procedures are provided in File S1.

### Eligibility criteria

Eligibility was defined according to the PICOS framework.

Population (P): children and adolescents aged 3–18 years with a clinical diagnosis of cerebral palsy confirmed by qualified physicians, with no restriction on sex.

Intervention (I): rehabilitation programs in which exercise was the main component. Exercise modality was not restricted and could include conventional, virtual, or immersive formats, provided that key parameters such as frequency, intensity, and duration (both per session and in total) were reported in sufficient detail to allow reproducibility.

Comparator (C): usual rehabilitation or health management (*e.g*., health education, standard care, or maintenance of daily lifestyle). Trials that compared different exercise modalities were also included to permit indirect comparisons within the network framework.

Outcome (O): Gross Motor Function Measure (GMFM), including both GMFM-66 and GMFM-88 versions, was used to assess changes in gross motor function.

Study design (S): Only RCTs were eligible.

### Exclusion criteria

Strict exclusion criteria were applied to ensure methodological validity and relevance. Review articles, protocols, commentaries, dissertations, conference abstracts, and non–peer-reviewed studies were excluded. Trials lacking sufficient statistical information for between-group comparison were omitted if additional data could not be obtained from authors. Studies scoring below 4 on the methodological quality assessment were considered at high risk of bias and excluded (Cashin & McAuley, 2020). Acute single-session exercise studies were not included. Interventions that were not primarily exercise-based, or in which the specific effect of exercise could not be isolated, were excluded. This included interventions relying mainly on robotic or high-technology devices, as well as hybrid formats such as music therapy, dance, equine-assisted therapy, yoga, or vibration-based training. Trials combining exercise with pharmacological treatment, electrical stimulation, or occupational therapy were also excluded when the independent contribution of exercise could not be determined. Finally, non-RCTs, studies without a control group, and trials lacking sufficient data for meta-analysis were omitted.

The inclusion and exclusion criteria were jointly developed by Qiang Xiong, Xing-liang Duan, and Peng-wei He; study selection based on these criteria was independently performed by Qiang Xiong and Xing-liang Duan, and any disagreements were resolved by Peng-wei He serving as the adjudicator.

### Data handling

During data management, two reviewers independently screened and extracted information according to predefined inclusion and exclusion criteria. Disagreements were first resolved through discussion; if consensus could not be reached, a third reviewer, not involved in the initial selection, adjudicated to ensure independence and fairness.

Extracted variables included:

Study characteristics: first author, year of publication, and country of origin. Participant characteristics: total sample size, mean age, and sex distribution. Intervention details: training modality, frequency, single-session duration, total intervention length, and corresponding metabolic equivalents (METs). Outcomes: pre- and post-intervention values for the primary endpoint. When means and standard deviations were not directly reported, they were estimated from medians, interquartile ranges, ranges, or sample sizes using established methods (Liu et al., 2022; Luo et al., 2018). If studies reported only standard errors, confidence intervals, or quartile deviations, values were converted to means and standard deviations with standard formulae to ensure consistency of pooled data (De Miguel-Rubio et al., 2023). Where multiple tools were used to assess the same outcome, only one was retained to avoid duplicate weighting in the synthesis.

### Quality appraisal

All included studies were independently assessed for methodological quality by two reviewers, following predetermined standards. Discrepancies were addressed through discussion; if unresolved, a third independent reviewer provided the final decision. The PEDro scale was used for appraisal, comprising 11 items: the first reflects external validity and is not scored, while the remaining 10 items generate a total score between 0 and 10. According to predefined thresholds, scores <4 were considered low quality (and excluded), 4–5 fair, 6–7 moderate, and ≥8 high quality (De Miguel-Rubio et al., 2023; Amer-Cuenca et al., 2023). The PEDro scale has been widely applied in physical therapy and rehabilitation research due to its ease of use and discriminative capacity, and is regarded as a reliable tool for assessing the design and reporting quality of RCTs. The PEDro quality assessment was independently conducted by Qiang Xiong and Xing-liang Duan, following the predefined scoring criteria, and any discrepancies in ratings were resolved through discussion with Peng-wei He, who acted as the adjudicator.

### Analytical paradigm and computational workflow

The primary aim was to delineate potential dose-response relationships between exercise prescriptions and motor skill improvement in individuals with cerebral palsy. Analyses were performed within a Bayesian random-effects network meta-regression (MBNMA) framework (Mawdsley et al., 2016). Prior to model estimation, the structure of the study network was inspected to confirm adequate connectivity, and the assumptions of transitivity and consistency were examined to ensure valid inference (Wheeler, Hickson & Waller, 2010).

Effect sizes were expressed as standardized mean differences (SMD, Hedges’ g) with 95% credible intervals (CrI), representing the relative efficacy of different exercise modalities and dose levels in enhancing motor skills (Etzioni & Kadane, 1995). Dose-effect scatterplots were first generated for visual inspection. Various nonlinear models-including Emax, restricted cubic splines, quadratic polynomials, and non-monotonic growth functions-were then tested (Pedder et al., 2019). Model selection considered the deviance information criterion (DIC), complexity, residual structure, and heterogeneity. The quadratic polynomial model demonstrated the most favorable performance across these metrics and was therefore chosen to characterize the final dose-response curves (Evans, 2019).

For standardization, exercise interventions were classified by modality, and metabolic equivalent values were assigned using the Compendium of Energy Expenditures for Youth (Butte et al., 2018). Weekly exercise dose was calculated as session duration × weekly frequency, expressed as METs·min/week (Wasfy & Baggish, 2016). To avoid instability from wide variation in reported energy expenditure, continuous dose values were categorized into intervals spanning 150–1,500 METs·min/week, thereby improving comparability and model convergence (Higgins et al., 2012). For studies that did not report training frequency or session duration, missing parameters were imputed using the mean values of available studies, while retaining all other reported data to ensure consistency in dose estimation. MET values were assigned using the Compendium of Energy Expenditures for Youth. When an exact match was unavailable, the closest activity was selected based on similarity in movement characteristics and reported intensity of the intervention. This standardized matching procedure ensured reproducible and comparable dose estimation across studies, consistent with recommended practice for applying the compendium to non-standard activities.

All statistical analyses were conducted in R (version 4.4.2). The MBNMAdose package was used for model construction and Bayesian inference, and ggplot2 was applied for visualization of dose-response curves.

### Evaluation of evidence certainty

Two reviewers independently assessed the certainty of evidence for each outcome. Disagreements were resolved by discussion and, if necessary, adjudicated by a third independent reviewer. Evidence certainty was graded using the Grading of Recommendations, Assessment, Development and Evaluation (GRADE) framework (Kavanagh, 2009), which considers methodological limitations, consistency, indirectness, imprecision, and risk of publication bias. Each key outcome was categorized into one of four levels: high, moderate, low, or very low. The GRADE evaluation of the overall certainty of evidence was independently conducted by Qiang Xiong and Xing-liang Duan in accordance with the GRADE guidelines, and any discrepancies in judgments were resolved through discussion with Peng-wei He, who served as the adjudicator.

## Results

### Literature search and study selection

**Figure 1:**
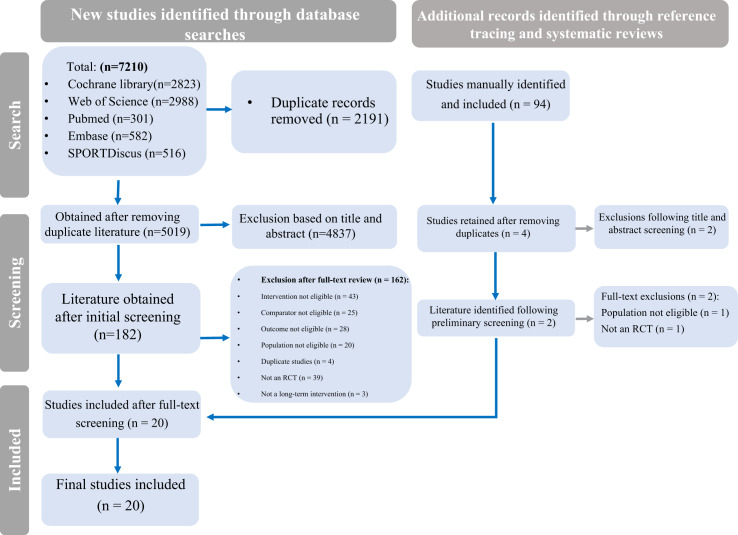
PRISMA flow diagram of study selection.

A systematic search was conducted on 10 August 2025 across five databases: Cochrane Library, Web of Science, PubMed, Embase, and SPORTDiscus. A total of 7,210 records were retrieved. After title and abstract screening, 182 articles underwent full-text review. Of these, 162 were excluded due to ineligible interventions, comparators, populations, outcomes, or study designs. Ultimately, 20 studies met the inclusion criteria. An additional 94 potential records were identified through reference checking and systematic review hand-searching, but none met eligibility after screening. Thus, 20 randomized controlled trials were included in the final analysis (Fig. 1).

### Characteristics of included studies

Twenty RCTs involving children and adolescents with cerebral palsy were included (Dodd, Taylor & Graham, 2003; Tsorlakis et al., 2004; Liao et al., 2007; Christiansen & Lange, 2008; Lee, Sung & Yoo, 2008; Bar-Haim et al., 2010; Fowler et al., 2010; Scholtes et al., 2010; Johnston et al., 2011; Chrysagis et al., 2012; Dimitrijević et al., 2012; Bryant et al., 2013; Chen et al., 2013; Grecco et al., 2013; Wang et al., 2013; Labaf et al., 2015; Curtis et al., 2018; Cho & Lee, 2020; Mohamed et al., 2025; Sakzewski et al., 2025). The mean age of participants was concentrated between 8 and 10 years. Most studies enrolled individuals classified as Gross Motor Function Classification System (GMFCS) levels I–III, reflecting good clinical homogeneity. All trials implemented structured exercise-based interventions. Modalities included body control training, treadmill or running programs, resistance training, aquatic therapy, cycling, functional exercise, and virtual reality-assisted training. Exercise intensity was quantified using metabolic equivalents (METs), with frequencies ranging from 2 to 5 sessions per week and session durations of 20–60 min. The primary outcome across studies was the Gross Motor Function Measure (GMFM), assessed using either GMFM-66 or GMFM-88. Detailed study characteristics are provided in Table S1.

### Methodological quality and risk of bias

Quality appraisal indicated that most trials maintained acceptable methodological rigor, with overall risk of bias considered limited. PEDro scores ranged from 5 to 8, with a mean of 6.7 (SD = 1.03). Notably, 85% of studies scored ≥6, suggesting a predominantly moderate-to-high quality evidence base (Table S2).

Due to the inherent nature of exercise interventions, blinding of participants and providers was generally unfeasible, representing a common limitation in this field. However, most studies employed blinded outcome assessment, effectively minimizing detection bias and strengthening the objectivity of measurements. Furthermore, randomization procedures and baseline comparability were clearly reported in the majority of trials, further enhancing credibility. Overall, despite some methodological constraints, the included studies were of sufficient quality to support robust evidence synthesis.

### Overall exercise dose and motor skill improvement

**Figure 2:**
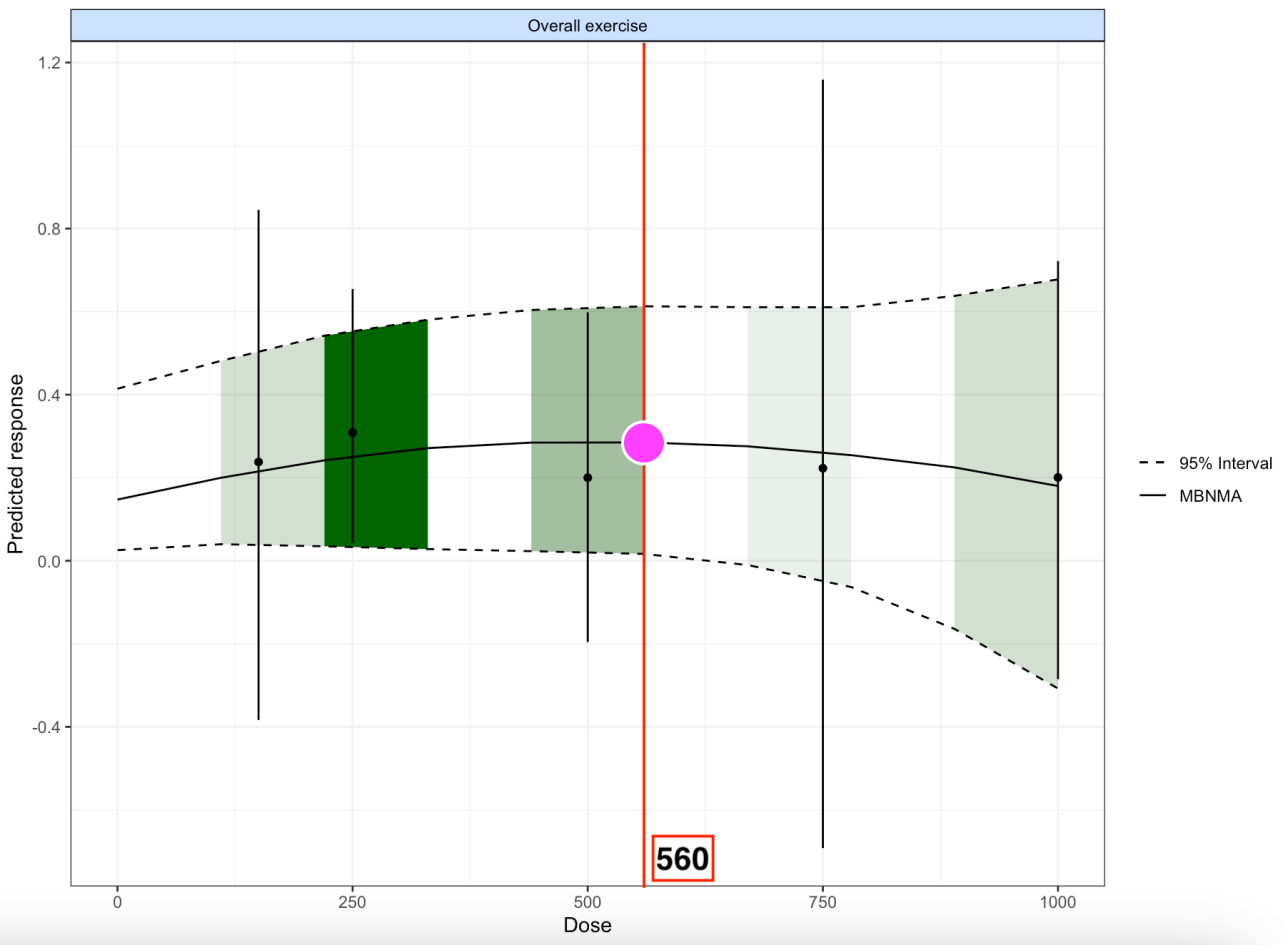
Dose-response relationship of overall exercise on motor skill improvement in individuals with cerebral palsy. Notes: The curve shows the nonlinear dose-response relationship between overall exercise and predicted motor skill outcomes in cerebral palsy. The black solid line represents the estimated mean effect based on MBNMA, and the dashed lines indicate the 95% credible interval. Green shaded areas highlight the distribution of available exercise doses, while the magenta circle and red vertical line mark the approximate optimal overall dose (560 MET-min/week).

Results from the MBNMA (Fig. 2) indicated that exercise interventions produced a significant but marginal effect on motor skill improvement in individuals with cerebral palsy (effect size = 0.295, 95% CrI 0.016–0.613). The dose-response curve showed that benefits increased with higher doses, peaking at approximately 560 METs·min/week. The most stable and clinically meaningful effects were observed within the moderate dose range of 200–600 METs·min/week. Beyond this threshold, the effect plateaued rather than continuing to rise. These findings suggest that moderate doses represent the key determinant of motor skill gains, whereas both insufficient and excessive doses may limit benefits. The pattern aligns with the inverted U-shaped dose-response relationship commonly described in exercise prescription research.

### Dose-response relationships by exercise modality

**Figure 3:**
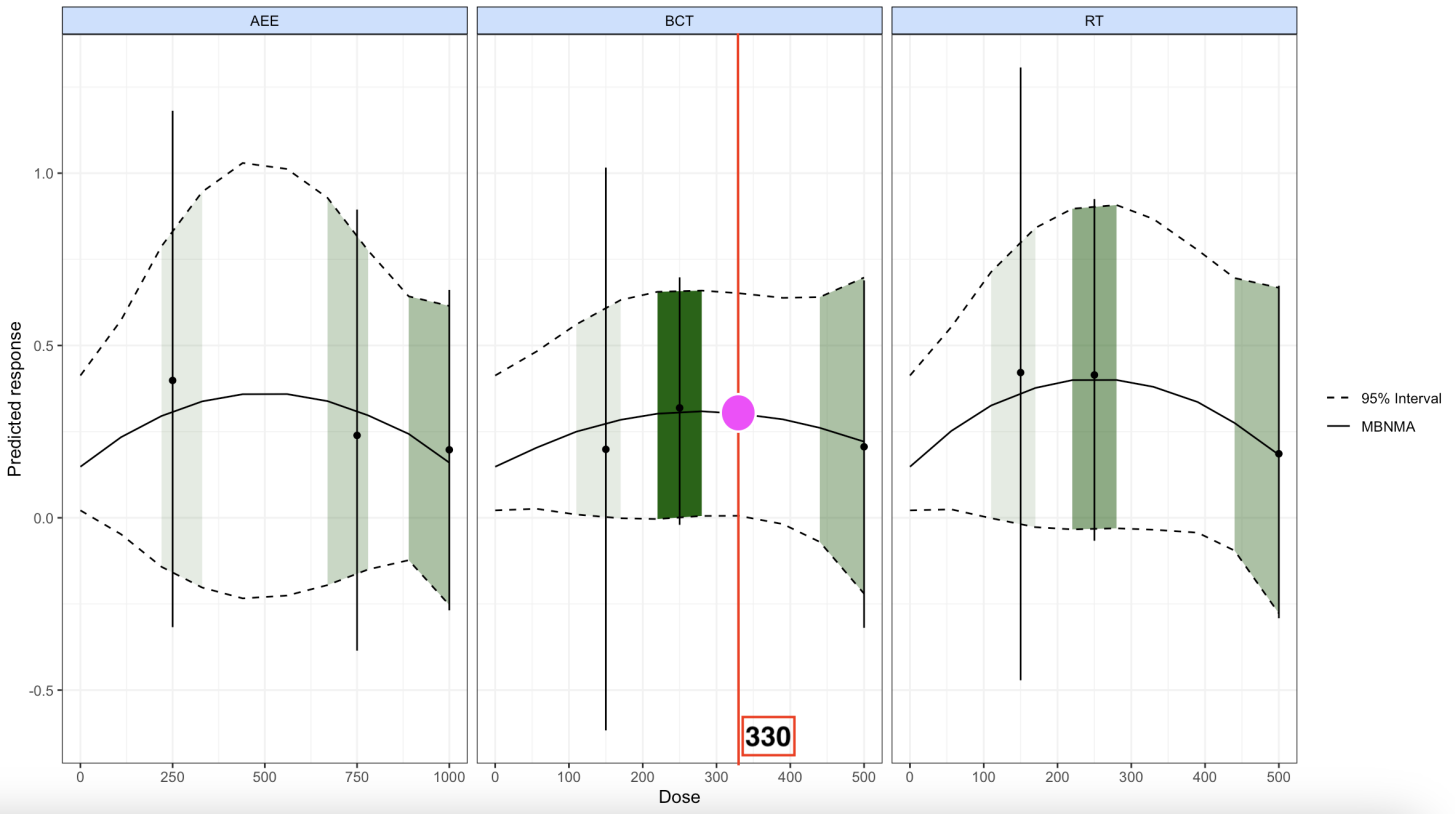
Dose–response relationships of aerobic exercise (AEE), body control training (BCT), and resistance training (RT) on motor skill outcomes in cerebral palsy. Notes: This figure presents the nonlinear dose-response associations of three exercise modalities (AEE, BCT, RT) with motor skill improvement in cerebral palsy. The black solid line indicates the mean predicted response, with dashed lines denoting the 95% credible interval. Green shaded regions represent the observed distribution of doses. The magenta circle and red vertical line identify the approximate optimal dose (330 MET-min/week), demonstrating that different modalities exhibit varying effect sizes and dose thresholds.

Because the number of studies for each specific modality was limited and heterogeneous, direct pooling could have compromised model stability. To enhance precision, interventions were grouped into three categories: aerobic exercise (AEE), body control training (BCT), and resistance training (RT) (Fig. 3). This classification reduced inter-modality heterogeneity and enabled clearer comparison of their respective dose-response patterns.

#### Aerobic exercise

AEE demonstrated a trend of “optimal effects at moderate doses with diminishing returns at higher levels.” Within 220–560 METs·min/week, effects gradually increased, peaking at 560 METs·min/week (mean = 0.365, 95% CrI −0.269 to 0.996). However, the credible interval crossed zero, indicating statistical non-significance. This result may reflect limited sample size, design variability, or substantial inter-individual differences in response to aerobic training among patients with CP. At doses ≥670 METs·min/week, the effect declined to near null, suggesting that higher volumes are not necessarily more beneficial.

#### Body control training

The dose-response curve for BCT was relatively stable, with an optimal range between 170 and 330 METs·min/week. At 330 METs·min/week, BCT produced a statistically significant effect (mean = 0.313, 95% CrI 0.014–0.666), at 330 METs·min/week, BCT produced a statistically credible effect (mean = 0.313, 95% CrI 0.014–0.666), making it the only modality for which the 95% CrI at the optimal dose did not cross zero. Compared with AEE and RT, the narrower credible intervals of BCT indicate greater reproducibility and clinical stability. This may be attributable to its direct targeting of postural control, balance, and core stability, features particularly relevant to the motor deficits of CP. Excessive doses (≥440 METs·min/week) attenuated the effect, further supporting the “moderate dose is best” pattern.

#### Resistance training

RT followed an inverted U-shaped curve, with the greatest improvement observed between 170 and 280 METs·min/week. At 280 METs·min/week, the effect approached significance (95% CrI −0.075 to 0.881). Beyond 390 METs·min/week, benefits declined, and at 500 METs·min/week the effect was negligible (mean = 0.182, 95% CrI −0.280 to 0.682). This suggests that RT at moderate doses may yield meaningful gains, but its efficacy is more sensitive to upper dose thresholds, where excessive load could lead to fatigue or impaired coordination.

Taken together, these findings highlight BCT at approximately 330 METs·min/week as the most reliable and clinically applicable prescription for motor rehabilitation in children and adolescents with CP.

### Certainty of evidence and publication bias

Across the 20 RCTs included, pooled results indicated that exercise interventions produced a moderate and clinically meaningful improvement in motor skills among children and adolescents with cerebral palsy. Overall methodological quality was acceptable, with a mean PEDro score of 6.7; more than three-quarters of the studies scored ≥6, indicating reasonable reliability in study design and execution. Moderate heterogeneity was observed (I^2^ = 49.68%), which may reflect differences in intervention modalities and participant characteristics.

Assessment of publication bias showed largely symmetrical funnel plots (Figs. S11, S12). Trim-and-fill analysis did not identify missing studies, although Egger’s regression reached the margin of significance (*p* = 0.05), suggesting that small-study effects or mild publication bias cannot be fully excluded. According to the GRADE framework (Table S7), the certainty of evidence was rated as moderate. While effect estimates were consistent and clinically relevant, limitations related to study size and potential bias introduced some uncertainty. Overall, the findings support the positive value of exercise interventions for motor skill rehabilitation in cerebral palsy, but emphasize the need for large-scale, multicenter trials with more rigorous methodology to further validate the dose-response relationship and guide precision-based prescriptions.

### Robustness analyses

To evaluate the reliability and generalizability of the dose-response findings, multiple robustness checks were conducted. First, alternative nonlinear functional forms were tested, and the quadratic model consistently provided the best fit across outcomes, thus selected for the final analysis. Second, re-estimation after excluding lower-quality trials yielded curves that preserved the same trends and optimal dose ranges, indicating that results were not driven by study quality. Third, leave-one-out sensitivity analysis showed no single trial exerted undue influence on the overall effect or dose estimates. Collectively, these procedures demonstrate that the established dose-response model was stable in functional fit, internally consistent, and resistant to bias from individual studies, thereby providing a reliable foundation for clinical interpretation and translation into practice.

Among the 20 included studies, only one trial (Sakzewski et al., 2025) did not report training frequency and session duration in a conventional weekly format. In the initial analysis, these missing parameters were imputed using the mean frequency and session duration derived from the other included studies in order to estimate weekly exercise dose. Recognizing that this procedure could potentially introduce bias into the dose–response modeling, an additional sensitivity analysis was conducted. Specifically, this study was removed from the dataset and the MBNMA was re-estimated. The resulting dose–response curves were nearly identical to those obtained in the primary analysis. The inverted U-shaped pattern was preserved, and the optimal dose range remained within 300–600 METs·min/week. This indicates that the overall findings were not influenced by the imputation of frequency and duration parameters for this study, confirming the robustness of the model to this potential source of uncertainty.

## Discussion

### Main findings, strengths, and differences from existing evidence

By synthesizing 20 RCTs, this study is among the first to apply a Bayesian dose–response framework to explore the nonlinear association between exercise dose and motor skill recovery in children with cerebral palsy. The modeling suggested that exercise interventions were associated with improvements across a wide range of doses, with the most consistent benefits observed at moderate levels (approximately 330–560 METs·min/week; peak effect size ≈0.295, 95% CrI 0.016–0.613). At higher doses (>670 METs·min/week), the magnitude of benefit appeared to plateau or become uncertain, underscoring the potential importance of dose appropriateness. These findings suggest that the value of rehabilitation may lie not only in whether exercise is delivered, but also in how much is prescribed (Brandao et al., 2018; Chen, Du & Zhu, 2025). Modality-specific analyses further indicated that aerobic exercise, body control training, and resistance training followed dose-dependent patterns, although with different apparent optimal ranges. Among them, body control training around 330 METs·min/week demonstrated the most reproducible and statistically credible effects. While these results provide useful insights into potential dose windows, they should be interpreted cautiously due to measurement and design heterogeneity across studies.

This study extends previous literature in three ways. First, prior reviews largely focused on whether exercise improves function, whereas the present analysis explored the shape of the dose–response relationship. Second, by translating intensity, frequency, and duration into a unified metric of metabolic equivalents (METs·min/week), this study enabled direct cross-trial comparisons and provided a quantifiable framework for understanding exercise dosage. Third, in addition to overall effects, modality-specific dose–response analyses offered a more nuanced perspective for individualized rehabilitation planning. From both clinical and scientific perspectives, the findings highlight the potential relevance of dose precision. Moderate exercise may facilitate neuroplasticity, enhance motor learning, and improve postural control and coordination, whereas excessive exercise may contribute to fatigue accumulation or reduced adherence, ultimately limiting long-term benefits (Pan, Zheng & He, 2025). Rather than proposing fixed prescriptions, the present findings provide an exploratory framework that may inform future research toward safer and more individualized rehabilitation strategies.

Interpreting the observed effect size in clinical terms is essential. A standardized mean difference of approximately 0.3 represents a small-to-moderate improvement in gross motor function, which is meaningful in the context of cerebral palsy rehabilitation. Previous research suggests that the minimal clinically important difference for GMFM generally ranges from 1.5 to 3.0 points, depending on baseline functional status and the instrument version used. In practical terms, the lower end of the identified dose range (around 330 METs·min/week) for body control training corresponds to roughly three 30-min sessions per week at moderate intensity, while the upper end (around 560 METs·min/week) would involve four to five sessions of similar duration. This range aligns well with typical rehabilitation schedules, indicating that the observed dose–response relationship offers a clinically relevant reference for planning training frequency and session length.

### Potential mechanisms through which exercise improves motor skills in cerebral palsy

The beneficial effects of exercise on motor skill development in individuals with CP are unlikely to result from a single pathway but rather from the convergence of multiple mechanisms. Central to this process is the enhancement of neuroplasticity. Neuroimaging and electrophysiological studies have demonstrated that systematic exercise training strengthens corticospinal tract connectivity and excitability, thereby improving the efficiency of signal transmission along motor pathways (Samsir et al., 2020). Task-specific repetitive practice further promotes synaptic remodeling, optimizes sensorimotor integration, and enhances the precision of motor planning and execution (Morgan et al., 2021).

In addition, improvements in musculoskeletal function provide the biomechanical foundation for motor skill gains. Progressive resistance training has been shown to increase muscle strength in children with CP without inducing adverse structural changes (Hanssen et al., 2022). By enhancing the contractile capacity of weak muscle groups and reducing abnormal co-contraction of antagonists caused by corticospinal tract damage, resistance training contributes to improved intermuscular balance. This, in turn, increases joint stability, reduces compensatory movement patterns during gait, and enhances the coordination and fine control of motor tasks (Hanssen et al., 2022; Eken et al., 2016).

Cardiorespiratory adaptation represents another important pathway. Regular exercise interventions have been shown to safely and effectively improve aerobic capacity in children and adolescents with CP. Enhanced cardiorespiratory fitness not only increases the efficiency of oxygen transport and utilization but also improves cerebral perfusion and oxygenation (Depiazzi et al., 2021; Soliman, Azab & Abdelbasset, 2022). These physiological adaptations create a more favorable neuro-metabolic environment, supporting both neural recovery and the acquisition of motor skills.

### Clinical implications, limitations, and future directions

#### Clinical implications

The principal contribution of this study lies in clarifying the dose-response relationship of exercise interventions for motor skill rehabilitation in children with cerebral palsy. Previous clinical practice has largely focused on selecting exercise modalities while neglecting systematic consideration of dose parameters such as intensity, frequency, duration, and cumulative exposure. As a result, rehabilitation prescriptions have often relied on convention and experience rather than evidence. By integrating data across trials, this study demonstrates that exercise dose not only determines whether a therapeutic effect emerges but also influences the magnitude and durability of improvement. These findings provide the basis for shifting rehabilitation prescriptions from a binary emphasis on participation toward precise, dose-oriented design.

From a functional standpoint, appropriate dosing enhances efficiency by maximizing improvements in GMFM scores within limited rehabilitation resources, while avoiding underdosing that yields negligible benefits or overdosing that leads to fatigue and reduced adherence. From a mechanistic perspective, effective activation of neuroplastic remodeling, musculoskeletal adaptation, and metabolic enhancement may depend on specific dose thresholds. Thus, dose precision is critical not only for short-term functional gains but also for long-term neural reorganization and skill maintenance. Importantly, recognition of the dose-response relationship provides quantifiable benchmarks for future guidelines and clinical pathways, enabling prescriptions to evolve from “exercise recommended” to “exercise at defined dose,” thereby advancing evidence-based, standardized, and individualized rehabilitation.

#### Limitations

Several limitations warrant consideration. The included trials were generally modest in sample size, and variation in exercise modalities and dosing parameters may have influenced the stability of the modeled dose–response curves. Although GMFM was used as the primary outcome, differences in instrument versions and assessment procedures across studies may have affected comparability. In particular, the use of standardized mean differences to combine outcomes from GMFM-66 and GMFM-88 assumes a degree of construct equivalence between the two versions that may not be fully justified, given their differences in structure, scaling, and measurement properties. In addition, the relatively short follow-up periods in most trials limited the ability to evaluate whether appropriate doses translate into sustained motor improvements over time.

A further methodological consideration concerns the use of standardized mean differences to combine outcomes from GMFM-66 and GMFM-88. While both instruments assess gross motor function in children with cerebral palsy, they differ in structure, scaling, and measurement properties. Pooling these measures therefore assumes a degree of construct equivalence that may not be fully warranted. Because the number of studies using each version separately was insufficient to support independent dose-response modeling, the present findings should be interpreted as exploratory with respect to the precise magnitude of dose effects. Future research with a larger evidence base should examine these instruments separately to determine whether the observed dose–response pattern is consistent across measurement approaches.

#### Future Directions

Future research should advance in three areas. Large-scale, multicenter RCTs using a standardized framework for dose reporting are needed to strengthen both robustness and comparability. Beyond functional outcomes such as GMFM, trials should incorporate imaging and physiological markers to elucidate the neural and muscular mechanisms underlying dose-response relationships. Finally, stratified analyses should be conducted to determine optimal dose ranges across functional subgroups, particularly children with higher GMFCS levels, to enable tiered and individualized prescriptions. Through such efforts, research on exercise dosing may transition from documenting functional improvement to achieving precision rehabilitation, ultimately advancing evidence-based and patient-tailored care for cerebral palsy.

## Conclusion

Using Bayesian model-based network meta-analysis, this study explored the dose–response pattern of exercise interventions for motor skill improvement in children with cerebral palsy. The findings suggested an inverted U-shaped trend, in which moderate exercise doses of approximately 330–560 METs·min/week appeared to be associated with more consistent benefits, whereas both lower and higher doses may be linked to reduced efficacy. Modality-specific analyses further suggested distinct dose characteristics, with body control training at around 330 METs·min/week showing the most reproducible and credible trend within the available evidence. These results highlight the potential value of moving beyond the question of whether to intervene toward more refined consideration of exercise dose in rehabilitation planning. However, given the limited sample size and modest effect magnitude, these dose patterns should be interpreted as indicative rather than definitive. Further validation through large-scale, multicenter trials is required before dose–response principles can be translated into clinical guidelines and evidence-based rehabilitation strategies.

## Supplemental Information

10.7717/peerj.21035/supp-1 Supplemental Information 1 Network geometry of overall exercise at different dose levels.

10.7717/peerj.21035/supp-2 Supplemental Information 2 Predicted dose–response relationship of overall exercise from MBNMA with 95% credible intervals.

10.7717/peerj.21035/supp-3 Supplemental Information 3 Rank probability distributions of overall exercise doses based on MCMC simulations.

10.7717/peerj.21035/supp-4 Supplemental Information 4 Posterior mean distributions of overall exercise effects across dose levels.

10.7717/peerj.21035/supp-5 Supplemental Information 5 Observed treatment responses of overall exercise at different dose levels on the link scale.

10.7717/peerj.21035/supp-6 Supplemental Information 6 Network geometry of different exercise modalities across dose levels. Notes: AEE, Aerobic Exercise; BCT, Body Control Training; RT, Resistance Training.

10.7717/peerj.21035/supp-7 Supplemental Information 7 Predicted dose–response relationship of different exercise modalities from MBNMA with 95% credible intervals.

10.7717/peerj.21035/supp-8 Supplemental Information 8 Rank probability distributions of different exercise modalities across dose levels based on MCMC simulations.

10.7717/peerj.21035/supp-9 Supplemental Information 9 Posterior mean distributions of different exercise modalities across dose levels.

10.7717/peerj.21035/supp-10 Supplemental Information 10 Observed treatment responses of different exercise modalities across dose levels on the link scale.

10.7717/peerj.21035/supp-11 Supplemental Information 11 Contour-enhanced funnel plot for publication bias assessment.

10.7717/peerj.21035/supp-12 Supplemental Information 12 Power-enhanced funnel plot for publication bias assessment.

10.7717/peerj.21035/supp-13 Supplemental Information 13 Supplementary Materials.

10.7717/peerj.21035/supp-14 Supplemental Information 14 PRISMA checklist.

10.7717/peerj.21035/supp-15 Supplemental Information 15 Audience.

## Additional Information and Declarations

### Data Availability

The following information was supplied regarding data availability:

This is a systematic review/meta-analysis.
