# Supplementary figures and images for: Overall and modality-specific exercise doses for motor skill improvement in cerebral palsy: a systematic review and Bayesian network dose-response meta-analysis
Source: PeerJ. 2026 Apr 8;14:e21035. doi: 10.7717/peerj.21035 (PMC13069938; doi:10.7717/peerj.21035)

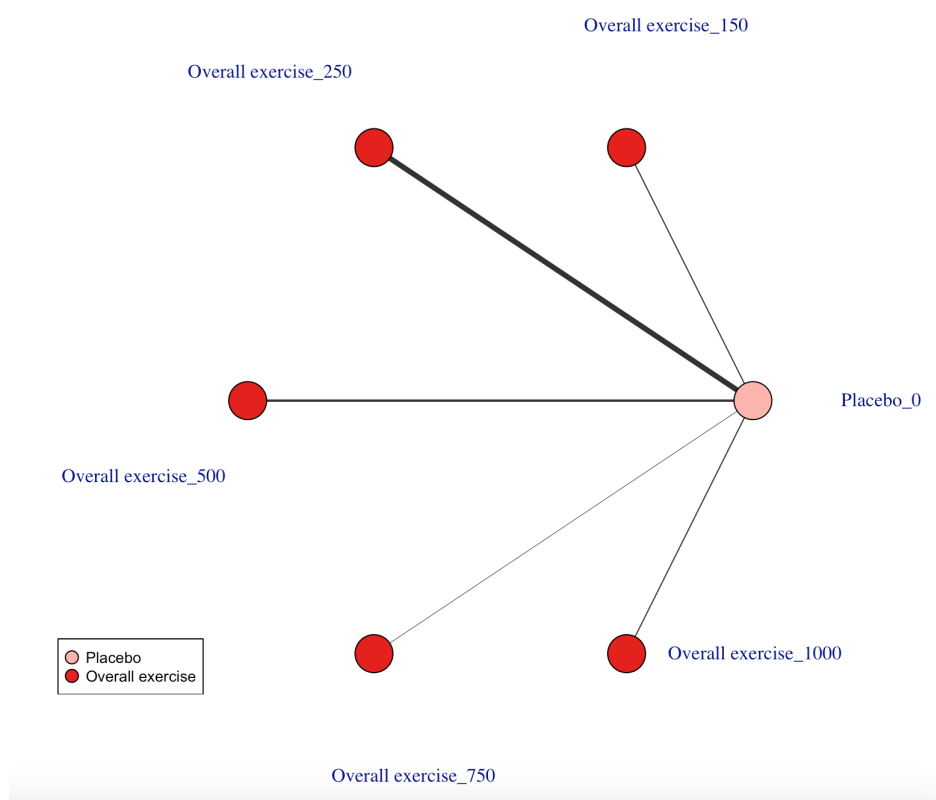

Supplement: Supplemental Information 1 [file peerj-14-21035-s001.pdf]

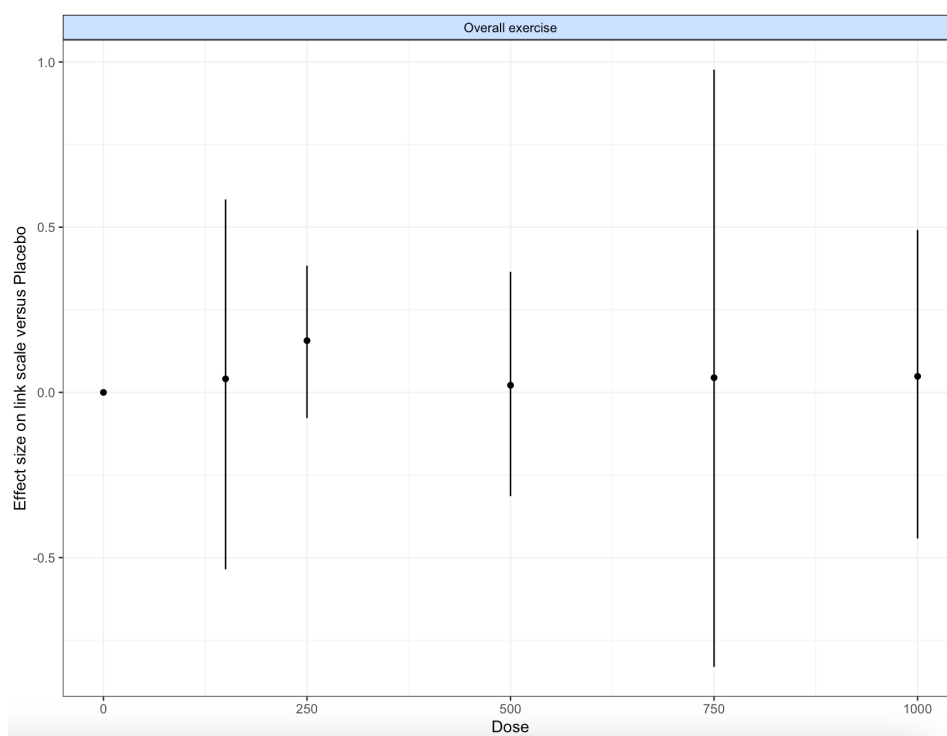

Supplement: Supplemental Information 2 [file peerj-14-21035-s002.pdf]

# Predictions

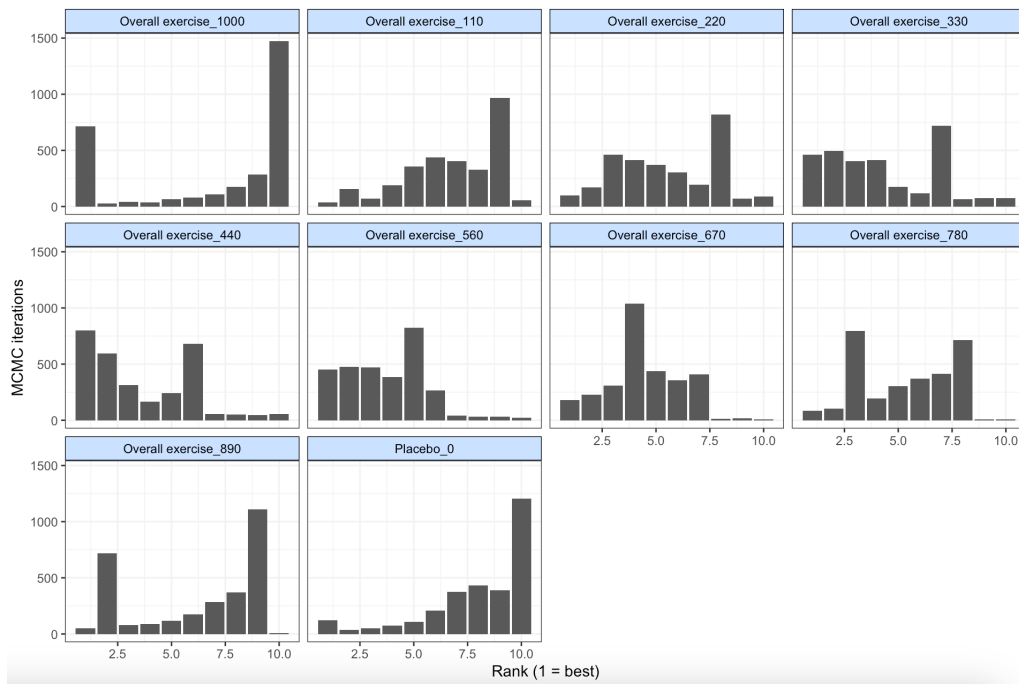

Supplement: Supplemental Information 3 [file peerj-14-21035-s003.pdf]

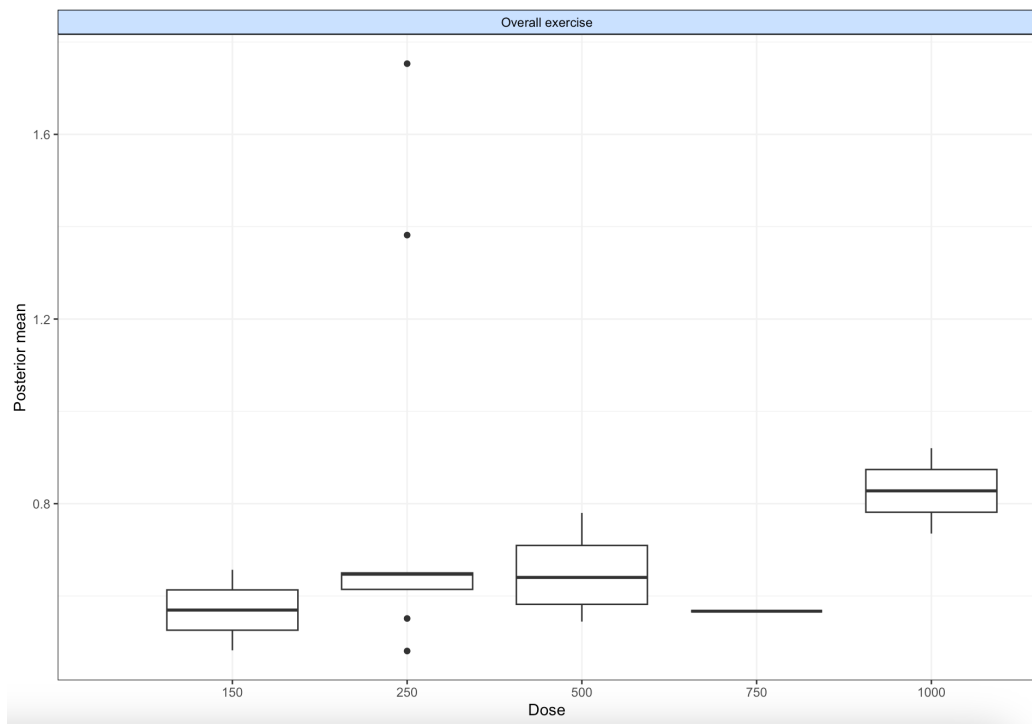

Supplement: Supplemental Information 4 [file peerj-14-21035-s004.pdf]

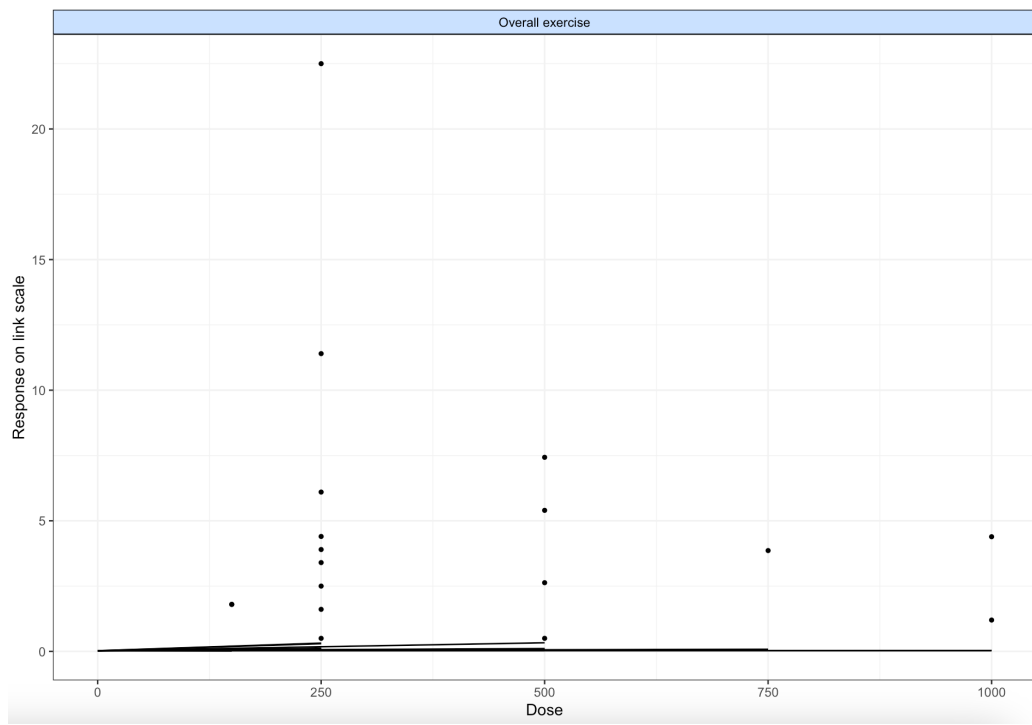

Supplement: Supplemental Information 5 [file peerj-14-21035-s005.pdf]

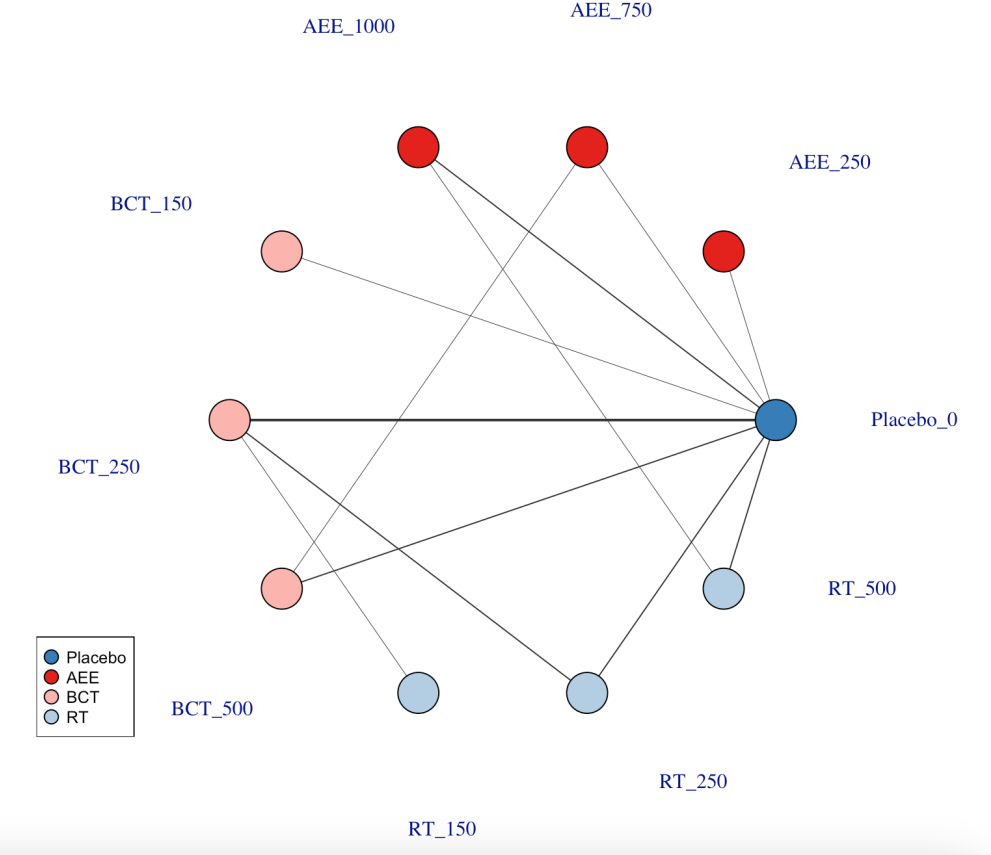

Supplement: Supplemental Information 6 — Notes: AEE, Aerobic Exercise; BCT, Body Control Training; RT, Resistance Training. [file peerj-14-21035-s006.pdf]

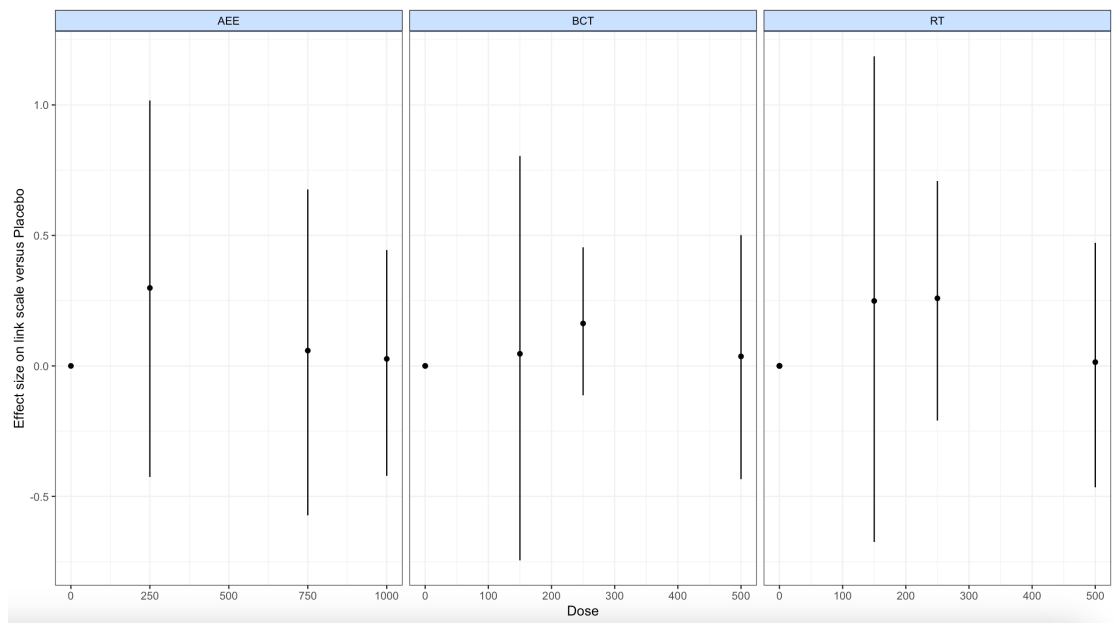

Supplement: Supplemental Information 7 [file peerj-14-21035-s007.pdf]

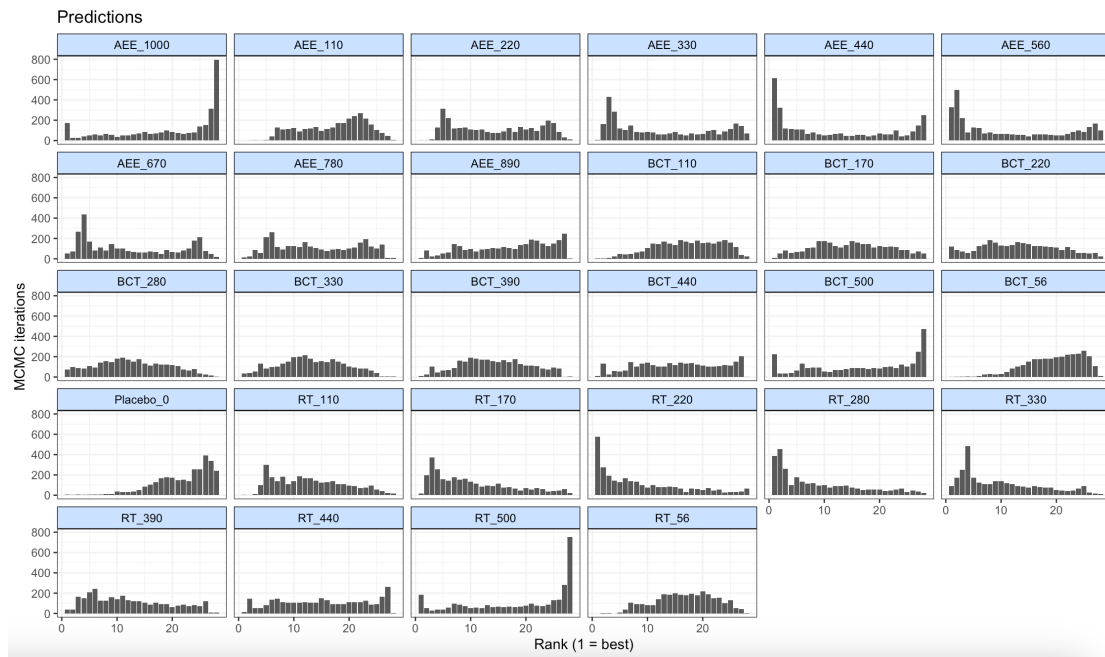

Supplement: Supplemental Information 8 [file peerj-14-21035-s008.pdf]

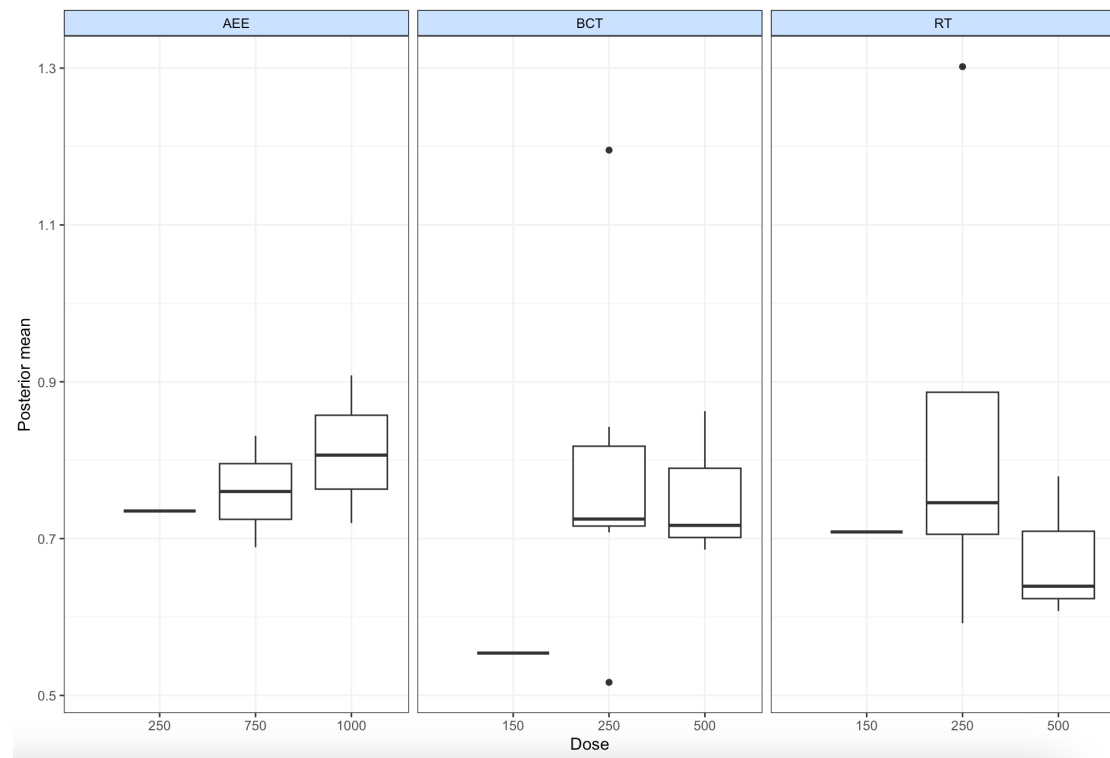

Supplement: Supplemental Information 9 [file peerj-14-21035-s009.pdf]

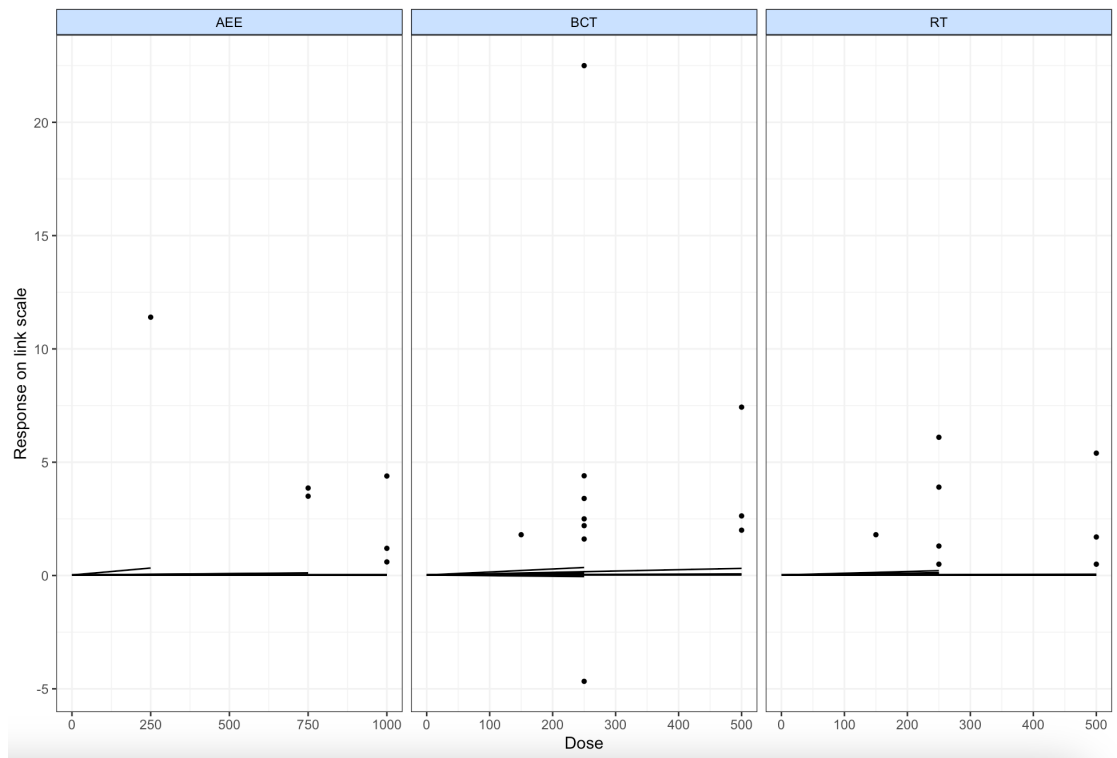

Supplement: Supplemental Information 10 [file peerj-14-21035-s010.pdf]

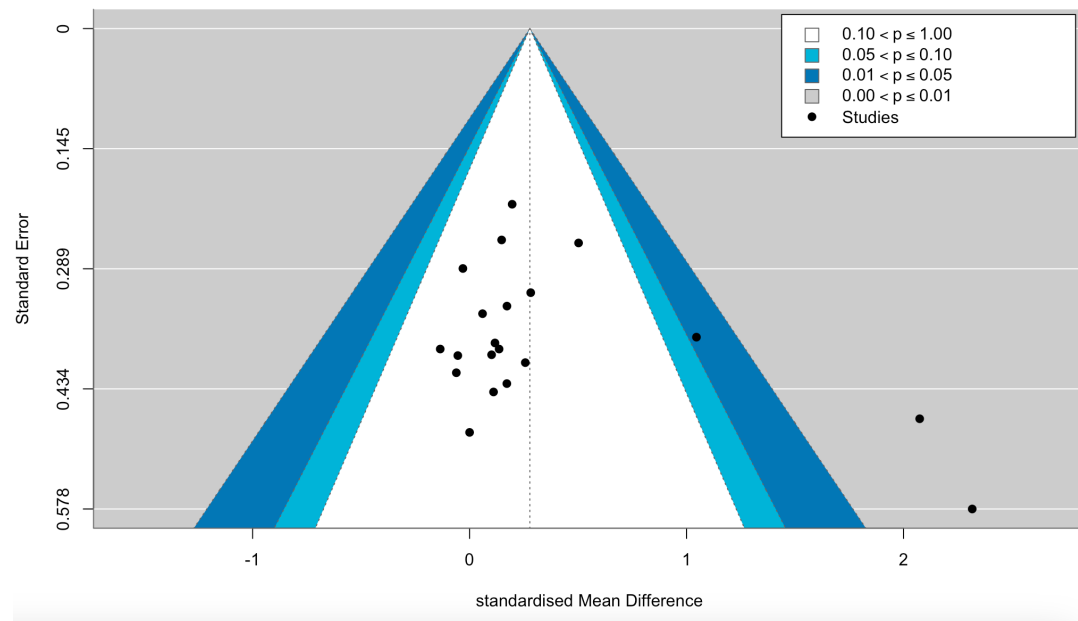

Supplement: Supplemental Information 11 [file peerj-14-21035-s011.pdf]

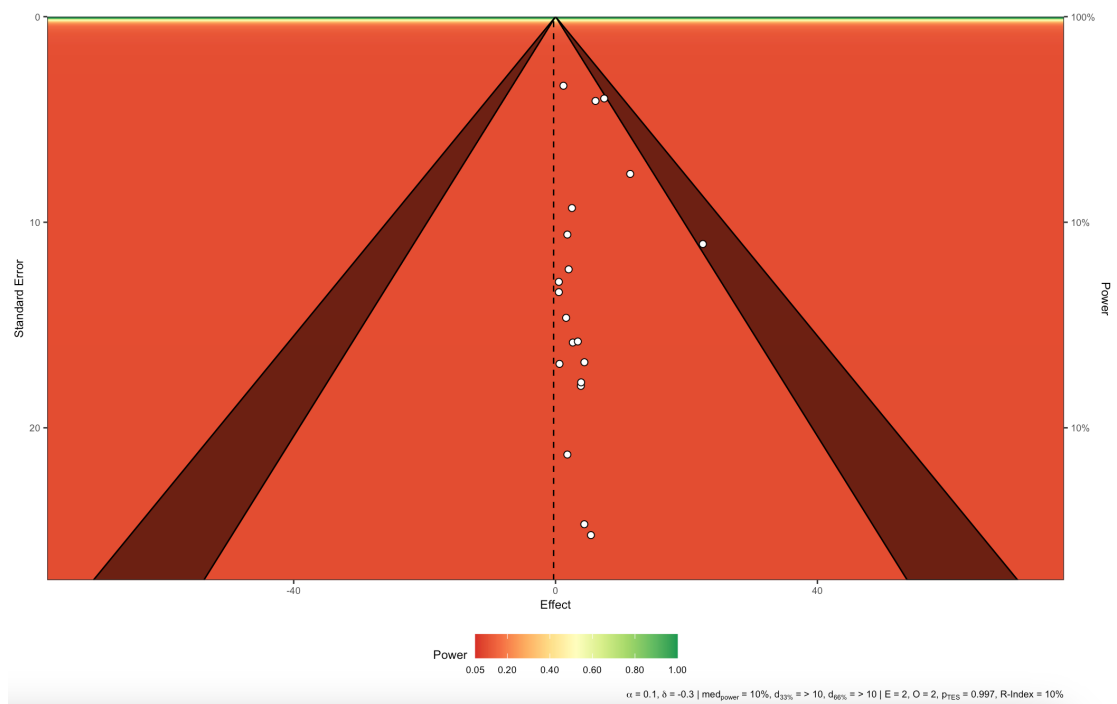

Supplement: Supplemental Information 12 [file peerj-14-21035-s012.pdf]
