# Supplementary material for: Overall and modality-specific exercise doses for motor skill improvement in cerebral palsy: a systematic review and Bayesian network dose-response meta-analysis
Source: PeerJ. 2026 Apr 8;14:e21035. doi: 10.7717/peerj.21035 (PMC13069938; doi:10.7717/peerj.21035)
Supplement: Supplemental Information 13 [file peerj-14-21035-s013.docx]

**Retrieval Strategies: (7210)**

**Cochrane Library (2823)**

**#1 Population**: (cerebral palsy OR diplegias OR cerebral palsies OR CP):ti, ab, kw

**#2 Intervention**: (dance OR strength OR hydrotherapy OR coordination OR sport OR flexibility OR martial art OR swim OR train OR virtual reality OR stretch OR yoga OR movement OR core control OR kinesiotherapy OR water sports OR activity OR stability OR aerobic OR mind-body exercise OR resistance OR activities OR sprint OR balance OR high intensity interval OR motor control OR endurance OR pilates OR walk OR functional training OR sports combined training OR physical OR exercise OR athletic OR tai chi OR run):ti, ab, kw

**#3 Outcomes**: (motor skill OR motor learning OR motor control OR skill acquisition OR skill learning OR perceptual motor skill OR fine motor skill OR gross motor skill OR motor performance OR skill transfer OR procedural learning OR psychomotor OR motor coordination OR motor development OR GMFM OR Gross Motor Function Measure):ti, ab, kw

**Search algorithm**: Trials matching "#4 - #1 AND #2 AND #3"

**Web of Science (2988)**

**#1 Population**: (cerebral palsy OR diplegias OR cerebral palsies OR CP) (Abstract)

**#2 Intervention**: (dance OR strength OR hydrotherapy OR coordination OR sport OR flexibility OR martial art OR swim OR train OR virtual reality OR stretch OR yoga OR movement OR core control OR kinesiotherapy OR water sports OR activity OR stability OR aerobic OR mind-body exercise OR resistance OR activities OR sprint OR balance OR high intensity interval OR motor control OR endurance OR pilates OR walk OR functional training OR sports combined training OR physical OR exercise OR athletic OR tai chi OR run) (Abstract)

**#3 Outcomes**: (motor skill OR motor learning OR motor control OR skill acquisition OR skill learning OR perceptual motor skill OR fine motor skill OR gross motor skill OR motor performance OR skill transfer OR procedural learning OR psychomotor OR motor coordination OR motor development OR GMFM OR Gross Motor Function Measure) (Abstract)

**Search algorithm**: #1 AND #2 AND #3 and Article (Document Types)

**PubMed (301)**

**#1 Population**: cerebral palsy[Title/Abstract] OR diplegias[Title/Abstract] OR cerebral palsies[Title/Abstract] OR CP[Title/Abstract]

**#2 Intervention**: dance[Title/Abstract] OR strength[Title/Abstract] OR hydrotherapy[Title/Abstract] OR coordination[Title/Abstract] OR sport[Title/Abstract] OR flexibility[Title/Abstract] OR martial art[Title/Abstract] OR swim[Title/Abstract] OR train[Title/Abstract] OR virtual reality[Title/Abstract] OR stretch[Title/Abstract] OR yoga[Title/Abstract] OR movement[Title/Abstract] OR core control[Title/Abstract] OR kinesiotherapy[Title/Abstract] OR water sports[Title/Abstract] OR activity[Title/Abstract] OR stability[Title/Abstract] OR aerobic[Title/Abstract] OR mind-body exercise[Title/Abstract] OR resistance[Title/Abstract] OR activities[Title/Abstract] OR sprint[Title/Abstract] OR balance[Title/Abstract] OR high intensity interval[Title/Abstract] OR motor control[Title/Abstract] OR endurance[Title/Abstract] OR pilates[Title/Abstract] OR walk[Title/Abstract] OR functional training[Title/Abstract] OR sports combined training[Title/Abstract] OR physical[Title/Abstract] OR exercise[Title/Abstract] OR athletic[Title/Abstract] OR tai chi[Title/Abstract] OR run[Title/Abstract]

**#3 Outcomes**: motor skill[Title/Abstract] OR motor learning[Title/Abstract] OR motor control[Title/Abstract] OR skill acquisition[Title/Abstract] OR skill learning[Title/Abstract] OR perceptual motor skill[Title/Abstract] OR fine motor skill[Title/Abstract] OR gross motor skill[Title/Abstract] OR motor performance[Title/Abstract] OR skill transfer[Title/Abstract] OR procedural learning[Title/Abstract] OR psychomotor[Title/Abstract] OR motor coordination[Title/Abstract] OR motor development[Title/Abstract] OR GMFM[Title/Abstract] OR Gross Motor Function Measure[Title/Abstract]

**Search algorithm**: #1 AND #2 AND #3 **Filters**: Clinical Trial

**Embase (582)**

**#1 Population**: cerebral palsy':ab,ti OR diplegias:ab,ti OR 'cerebral palsies':ab,ti OR cp:ab,ti

**#2 Intervention**: dance:ab,ti OR strength:ab,ti OR hydrotherapy:ab,ti OR coordination:ab,ti OR sport:ab,ti OR flexibility:ab,ti OR 'martial art':ab,ti OR swim:ab,ti OR train:ab,ti OR 'virtual reality':ab,ti OR stretch:ab,ti OR yoga:ab,ti OR movement:ab,ti OR 'core control':ab,ti OR kinesiotherapy:ab,ti OR 'water sports':ab,ti OR activity:ab,ti OR stability:ab,ti OR aerobic:ab,ti OR 'mind-body exercise':ab,ti OR resistance:ab,ti OR activities:ab,ti OR sprint:ab,ti OR balance:ab,ti OR 'high intensity interval':ab,ti OR 'motor control':ab,ti OR endurance:ab,ti OR pilates:ab,ti OR walk:ab,ti OR 'functional training':ab,ti OR 'sports combined training':ab,ti OR physical:ab,ti OR exercise:ab,ti OR athletic:ab,ti OR 'tai chi':ab,ti OR run:ab,ti

**#3 Outcomes**: 'motor skill':ab,ti OR 'motor learning':ab,ti OR 'motor control':ab,ti OR 'skill acquisition':ab,ti OR 'skill learning':ab,ti OR 'perceptual motor skill':ab,ti OR 'fine motor skill':ab,ti OR 'gross motor skill':ab,ti OR 'motor performance':ab,ti OR 'skill transfer':ab,ti OR 'procedural learning':ab,ti OR psychomotor:ab,ti OR 'motor coordination':ab,ti OR 'motor development':ab,ti OR gmfm:ab,ti OR 'gross motor function measure':ab,ti

**Search algorithm**: #1 AND #2 AND #3 AND ([controlled clinical trial]/lim OR [randomized controlled trial]/lim)

**SPORTDiscus (516)**

**#1 Population**: XB (cerebral palsy OR diplegias OR cerebral palsies OR CP)

**#2 Intervention**: XB (dance OR strength OR hydrotherapy OR coordination OR sport OR flexibility OR martial art OR swim OR train OR virtual reality OR stretch OR yoga OR movement OR core control OR kinesiotherapy OR water sports OR activity OR stability OR aerobic OR mind-body exercise OR resistance OR activities OR sprint OR balance OR high intensity interval OR motor control OR endurance OR pilates OR walk OR functional training OR sports combined training OR physical OR exercise OR athletic OR tai chi OR run)

**#3 Outcomes**: XB (motor skill OR motor learning OR motor control OR skill acquisition OR skill learning OR perceptual motor skill OR fine motor skill OR gross motor skill OR motor performance OR skill transfer OR procedural learning OR psychomotor OR motor coordination OR motor development OR GMFM OR Gross Motor Function Measure)

**Search algorithm**: #1 AND #2 AND #3

Supplementary Table 1 Characteristics of included studies

| **Source** | **Sample size（E/C）** | **Gender (E/C(M,F))** | **Age, Mean±SD** | **Intervention type** | **Code (Mets）** | **Weeks/Frequency per week/Duration-min** | **Intervention type** | **Weeks/Frequency per week/Duration-min** | Outcome |
| --- | --- | --- | --- | --- | --- | --- | --- | --- | --- |
| Dodd KJ 2003 | 11/10 | 4,7 / 6,4 | 13.1±3.1 | Resistance training | 851404 (3.2) | 6/3/40 | Care-as-usual | 6/NA/NA | GMFM-88 |
| Tsorlakis N 2004 | 17/17 | 11,6 / 11,6 | 7.1±3.6 / 7.5±3.7 | Neurodevelopmental treatment | 201602 (2.2) | 16/5/50 | Care-as-usual | 16/2/50 | GMFM-88 |
| Liao 2007 | 10/10 | 7,3/ 5,5 | 8.0±2.1 / 7.7±2.4 | Resistance training | 851402 (2.2) | 6/3/40 | Care-as-usual | 6/3/40 | GMFM-88 |
| Lee 2008 | 9/8 | 4 ,5 / 6 ,2 | 6.3±2.1 / 6.3±2.9 | Resistance training | 851402 (2.2) | 5/3/60 | Care-as-usual | 5/NA/NA | GMFM |
| Christiansen 2008 | 10/14 | 16, 9 | 2-8 | Sensory integration training | 155402 (4.5) | 30/4/45 | Care-as-usual | 30/4/45 | GMFM-66 |
| Fowler 2010 | 31/31 | 18,13/11,20 | 11.1/11.6 | Cycling training | 251203 (5.6) | 12/3/60 | Care-as-usual | 12/NA/NA | GMFM-66 |
| Scholtes 2010 | 25/24 | 13, 12/16, 8 | 10.3± 1.1/ 10.2 ± 2.2 | Resistance training | 851403 (2.7) | 12 /3/60 | Care-as-usual | 12/NA/NA | GMFM-66 |
| Bar-Haim 2010 | 39/39 | 19, 20/25,14 | 8.8 ± 1.72 / 8.9 ± 1.72 | Neurodevelopmental treatment | 201602 (2.2) | 12 /3/ 60 | Care-as-usual | 12 /NA/ 60 | GMFM-66 |
| Johnston 2011 | 14/12 | NA | 9.6 ± 2.0 / 9.8 ± 2.1 | Running training | 601203 (7), 851403 (2.7) | 12/5/30 | Resistance training | 12/5/30 | GMFM |
| Dimitrijević 2012 | 14/13 | 10,4/7,6 | 9.21± 2.45 / 9.92 ± 2.32 | [Aquatic exercise](https://www.baidu.com/s?rsv_idx=1&wd=Aquatic%20Exercise%E7%BF%BB%E8%AF%91&fenlei=256&usm=4&ie=utf-8&rsv_pq=e19dc7dc001a461b&oq=%E6%B0%B4%E4%B8%AD%E8%BF%90%E5%8A%A8%E8%8B%B1%E6%96%87&rsv_t=16fbJMxK7J1wxMyP8Z/54Xhc2t1pUtl6BNZvdnb0h0Qi1+qmBpRMnRr23LQ&sa=re_fy_huisou) | 751203 (8.3) | 6/2/55 | Care-as-usual | 6/2/55 | GMFM-88 |
| Chrysagis 2012 | 11/11 | NA | 15.90±1.97/16.09±1.51) | Running training | 601205 (7.5) | 12/3/30 | Care-as-usual | 12/3/30 | GMFM |
| Grecco 2013 | 16 / 17 | 6,10 / 9,8 | 6.8±2.6 / 6.0±1.5 | Running training | 601202 (5.6), 801202 (2.8) | 7/2/30 | Walking training | 7/2/30 | GMFM-88 |
| Chen 2013 | 13 / 14 | 9,4 / 9,5 | 8.7±2.1/8.6±2.2 | Virtual cycling training | 151002 (6), 601203 (7) | 12/3/30 | Running training | 12/3/30 | GMFM-66 |
| Wang 2013 | 18 / 18 | 12,6/15,3 | 9.00±1.99/8.98±2.61 | Combined motor-cognitive training | 151202 (2.7), 851403 (2.7) | 6/3/25 | Resistance training | 6/3/25 | GMFM-88 |
| Bryant 2013 | 11/12/12 | 6,5 /3,9,5,7 | 14.3±1.9/13.5±2.6/13.8±2.3 | Cycling training, Running training | 251204 (5.8), 601204 (7.3) | 6/3/30 | Care-as-usual | 6/NA/NA | GMFM-66 |
| Labaf 2015 | 15 / 13 | 7,8 / 7,6 | 4.9/4.4 | Neurodevelopmental treatment | 201602 (2.2) | 6/3/30 | Care-as-usual | 6/3/30 | GMFM-88 |
| Curtis 2017 | 14/14 | 11,3 / 6,7 | 8.5±4.0 / 8.5±4.0 | Body control training | 201602 (2.2), 851002(3.0) | 8/5/30 | Functional training | 8/NA/NA | GMFM |
| Cho 2020 | 13 / 12 | 4,9 / 8,4 | 5.54±1.81 / 7.17±2.17 | Resistance training | 851402 (2.2), 851002(3.0) | 6/3/30 | Functional training | 6/3/30 | GMFM-88 |
| Sakzewski 2025 | 46 / 44 | 25,21 / 29,15 | 10.3±2.9/10.3±3.2 | Functional training | 851003 (3.3) | 2/NA/NA | Care-as-usual | 2/5/NA | GMFM-66 |
| Mohamed 2025 | 20 / 20 | 28/12 | 6.2±1.87 / 5.4±0.97 | Body control training | 201602 (2.2) | 12/3/60 | Care-as-usual | 12/3/30 | GMFM-88 |

Notes: M, male; F, female; E, experimental group; C, control group; NA, not applicable; GMFM. Gross Motor Function Measure; SD, standard deviation.

Supplementary Table 2 Methodological quality of included studies assessed by the PEDro scale

| **Study** | **A1** | **A2** | **A3** | **A4** | **A5** | **A6** | **A7** | **A8** | **A9** | **A10** | **A11** | **Total score (out of 10)** |
| --- | --- | --- | --- | --- | --- | --- | --- | --- | --- | --- | --- | --- |
| Dodd KJ 2003 | 1 | 1 | 1 | 0 | 0 | 0 | 1 | 1 | 0 | 1 | 1 | 6 |
| Tsorlakis N 2004 | 1 | 1 | 0 | 1 | 0 | 0 | 1 | 1 | 0 | 1 | 1 | 6 |
| Liao 2007 | 1 | 1 | 1 | 1 | 1 | 0 | 1 | 0 | 1 | 1 | 1 | 8 |
| Lee 2008 | 1 | 1 | 1 | 1 | 1 | 0 | 1 | 0 | 1 | 1 | 1 | 8 |
| Christiansen 2008 | 1 | 1 | 0 | 1 | 0 | 0 | 1 | 1 | 1 | 1 | 1 | 7 |
| Fowler 2010 | 1 | 1 | 1 | 1 | 0 | 0 | 1 | 1 | 0 | 1 | 1 | 7 |
| Scholtes 2010 | 1 | 1 | 1 | 1 | 0 | 0 | 1 | 1 | 0 | 1 | 1 | 7 |
| Bar-Haim 2010 | 1 | 1 | 1 | 1 | 0 | 0 | 0 | 1 | 0 | 1 | 1 | 6 |
| Johnston 2011 | 1 | 1 | 1 | 1 | 0 | 0 | 1 | 1 | 0 | 1 | 1 | 7 |
| Dimitrijević 2012 | 1 | 1 | 1 | 1 | 0 | 0 | 0 | 1 | 0 | 1 | 1 | 6 |
| Chrysagis 2012 | 1 | 1 | 1 | 1 | 0 | 0 | 1 | 1 | 0 | 1 | 1 | 7 |
| Grecco 2013 | 1 | 1 | 1 | 1 | 1 | 0 | 1 | 0 | 1 | 1 | 1 | 8 |
| Chen 2013 | 1 | 1 | 0 | 1 | 0 | 0 | 0 | 1 | 0 | 1 | 1 | 5 |
| Wang 2013 | 1 | 1 | 1 | 1 | 0 | 0 | 1 | 1 | 1 | 1 | 1 | 8 |
| Labaf 2015 | 1 | 1 | 0 | 1 | 0 | 0 | 0 | 1 | 0 | 1 | 1 | 5 |
| Curtis 2017 | 1 | 1 | 0 | 0 | 0 | 0 | 1 | 0 | 1 | 1 | 1 | 5 |
| Ballington 2018 | 1 | 1 | 0 | 1 | 0 | 0 | 1 | 1 | 0 | 1 | 1 | 6 |
| Cho 2020 | 1 | 1 | 0 | 1 | 0 | 0 | 1 | 1 | 1 | 1 | 1 | 7 |
| Sakzewski 2025 | 1 | 1 | 1 | 1 | 0 | 0 | 1 | 0 | 1 | 1 | 1 | 7 |
| Mohamed 2025 | 1 | 1 | 1 | 1 | 0 | 0 | 1 | 1 | 1 | 1 | 1 | 8 |

Notes: A1, participant selection criteria specified; A2, random assignment of participants to groups; A3, hidden assignment; A4, groups were similar at baseline; A5, all participants were blind; A6, all therapists were blind; A7, all assessors were blind; A8, measurement of at least one of the main outcomes was obtained from more than 85% of the participants. A9, intention-to-treat analysis was conducted; A10, results of statistical comparisons between groups for at least one main outcome were reported; A11, the study reported point and variability measures for at least one main outcome; 1, the entry is satisfied; 0, the entry is not satisfied.

Supplementary Table 3. Dose-Response Comparisons of Exercise at Different Levels: Direct, Indirect, and MBNMA Estimates

| **Comparison** | **p-value** | **Median** | **0.025** | **0.975** |
| --- | --- | --- | --- | --- |
| Overall exercise_1000 vs Placebo_0 | 0.631 |  |  |  |
| -> direct |  | 0.015 | -0.404 | 0.471 |
| -> indirect |  | 0.239 | -0.254 | 0.743 |
| -> MBNMA |  | 0.12 | -0.207 | 0.456 |
|  |  |  |  |  |
| Overall exercise_750 vs Placebo_0 | 0.498 |  |  |  |
| -> direct |  | 0.088 | -0.805 | 0.873 |
| -> indirect |  | 0.096 | -0.182 | 0.365 |
| -> MBNMA |  | 0.09 | -0.155 | 0.342 |
|  |  |  |  |  |
| Overall exercise_500 vs Placebo_0 | 0.682 |  |  |  |
| -> direct |  | 0.013 | -0.33 | 0.364 |
| -> indirect |  | 0.063 | -0.139 | 0.247 |
| -> MBNMA |  | 0.06 | -0.104 | 0.228 |
|  |  |  |  |  |
| Overall exercise_250 vs Placebo_0 | 0.328 |  |  |  |
| -> direct |  | 0.157 | -0.066 | 0.383 |
| -> indirect |  | 0.013 | -0.073 | 0.096 |
| -> MBNMA |  | 0.03 | -0.052 | 0.114 |
|  |  |  |  |  |
| Overall exercise_150 vs Placebo_0 | 0.174 |  |  |  |
| -> direct |  | 0.053 | -0.553 | 0.596 |
| -> indirect |  | 0.016 | -0.029 | 0.067 |
| -> MBNMA |  | 0.018 | -0.031 | 0.068 |

Supplementary Table 4. Modality-to-Modality Comparisons of Exercise at Different Dose Levels: Direct, Indirect, and MBNMA Estimates

| **Comparison** | **p-value** | **Median** | **2.50%** | **97.50%** |
| --- | --- | --- | --- | --- |
| RT_250 vs BCT_250 | 0.644 |  |  |  |
| -> direct |  | -0.015 | -0.56 | 0.484 |
| -> indirect |  | 0.005 | -0.258 | 0.251 |
| -> MBNMA |  | -0.003 | -0.241 | 0.217 |
| RT_150 vs BCT_250 | 0.361 |  |  |  |
| -> direct |  | 0.072 | -0.752 | 0.861 |
| -> indirect |  | -0.02 | -0.2 | 0.163 |
| -> MBNMA |  | -0.024 | -0.208 | 0.151 |
| RT_500 vs AEE_1000 | 0.824 |  |  |  |
| -> direct |  | -0.01 | -0.794 | 0.818 |
| -> indirect |  | 0.024 | -0.522 | 0.587 |
| -> MBNMA |  | 0.028 | -0.424 | 0.451 |
| BCT_500 vs AEE_750 | 0.696 |  |  |  |
| -> direct |  | -0.016 | -0.813 | 0.869 |
| -> indirect |  | -0.076 | -0.484 | 0.372 |
| -> MBNMA |  | -0.057 | -0.432 | 0.326 |
| BCT_150 vs Placebo_0 | 0.238 |  |  |  |
| -> direct |  | 0.034 | -0.784 | 0.771 |
| -> indirect |  | 0.029 | -0.063 | 0.114 |
| -> MBNMA |  | 0.032 | -0.058 | 0.129 |
| AEE_250 vs Placebo_0 | 0.201 |  |  |  |
| -> direct |  | 0.263 | -0.432 | 1.039 |
| -> indirect |  | 0.016 | -0.076 | 0.103 |
| -> MBNMA |  | 0.018 | -0.063 | 0.11 |

Supplementary Table 5. Summary of Dose-Response Predictions for Overall Exercise Based on MBNMA

| **agent** | **dose** | **mean** | **sd** | **2.50%** | **25%** | **50%** | **75%** | **97.50%** |
| --- | --- | --- | --- | --- | --- | --- | --- | --- |
| Placebo | 0 | 0.1697072 | 0.1046899 | 0.02552002 | 0.09210865 | 0.147404 | 0.2289056 | 0.4144075 |
| Overall exercise | 0 | 0.1697072 | 0.1046899 | 0.02552002 | 0.09210865 | 0.147404 | 0.2289056 | 0.4144075 |
| Overall exercise | 110 | 0.2180933 | 0.1133598 | 0.03990321 | 0.13463709 | 0.1998613 | 0.2896812 | 0.4814925 |
| Overall exercise | 220 | 0.2548929 | 0.1293881 | 0.03477692 | 0.16151318 | 0.2418974 | 0.338449 | 0.5420782 |
| Overall exercise | 330 | 0.280106 | 0.142933 | 0.02813554 | 0.17813328 | 0.2712958 | 0.3723726 | 0.58062 |
| Overall exercise | 440 | 0.2937326 | 0.1512373 | 0.02316593 | 0.18670841 | 0.2846122 | 0.3927236 | 0.6040622 |
| Overall exercise | 560 | 0.2953837 | 0.1558339 | 0.01642695 | 0.18559497 | 0.2850291 | 0.3984346 | 0.6129181 |
| Overall exercise | 670 | 0.284784 | 0.1608082 | -0.01025123 | 0.17414768 | 0.2757182 | 0.3925573 | 0.6106725 |
| Overall exercise | 780 | 0.2625978 | 0.1740491 | -0.06329 | 0.14122726 | 0.2543456 | 0.3794383 | 0.6105782 |
| Overall exercise | 890 | 0.2288252 | 0.2031863 | -0.16421815 | 0.09145879 | 0.2248893 | 0.3650611 | 0.6379463 |
| Overall exercise | 1000 | 0.183466 | 0.2523737 | -0.30778706 | 0.01484954 | 0.1800824 | 0.3561898 | 0.6777244 |

Supplementary Table 6. Dose-Response Predictions of Different Exercise Modalities Based on MBNMA

| **agent** | **dose** | **mean** | **sd** | **0.025** | **0.25** | **0.5** | **0.75** | **0.975** |
| --- | --- | --- | --- | --- | --- | --- | --- | --- |
| Placebo | 0 | 0.1686637 | 0.103736 | 0.024024695 | 0.091307291 | 0.1480913 | 0.2265325 | 0.4250405 |
| Aerobic Exercise | 0 | 0.1686637 | 0.103736 | 0.024024695 | 0.091307291 | 0.1480913 | 0.2265325 | 0.4250405 |
| Aerobic Exercise | 110 | 0.2468178 | 0.1647942 | -0.058910246 | 0.136967492 | 0.2410714 | 0.3529805 | 0.5868106 |
| Aerobic Exercise | 220 | 0.3055753 | 0.2453428 | -0.163147907 | 0.14948247 | 0.3046812 | 0.4674298 | 0.7820862 |
| Aerobic Exercise | 330 | 0.3449362 | 0.3019241 | -0.246962711 | 0.155525074 | 0.3457527 | 0.5443415 | 0.9378643 |
| Aerobic Exercise | 440 | 0.3649004 | 0.3289421 | -0.278171984 | 0.158862404 | 0.36566 | 0.5812427 | 1.0142603 |
| Aerobic Exercise | 560 | 0.3645579 | 0.3244121 | -0.268950704 | 0.157614727 | 0.3688533 | 0.5757841 | 0.9958129 |
| Aerobic Exercise | 670 | 0.3439657 | 0.2917643 | -0.216136641 | 0.159735006 | 0.3456803 | 0.535428 | 0.9208754 |
| Aerobic Exercise | 780 | 0.3039768 | 0.2405085 | -0.170321873 | 0.147160797 | 0.3015344 | 0.4596975 | 0.7824358 |
| Aerobic Exercise | 890 | 0.2445914 | 0.1990409 | -0.134460039 | 0.114299702 | 0.239027 | 0.3734217 | 0.6594049 |
| Aerobic Exercise | 1000 | 0.1658094 | 0.2313594 | -0.274594551 | 0.006421005 | 0.1600478 | 0.317094 | 0.6395911 |
| Body Control Training | 0 | 0.1686637 | 0.103736 | 0.024024695 | 0.091307291 | 0.1480913 | 0.2265325 | 0.4250405 |
| Body Control Training | 56 | 0.2211072 | 0.1196193 | 0.027730036 | 0.135622238 | 0.2066261 | 0.2910046 | 0.4982527 |
| Body Control Training | 110 | 0.2608566 | 0.1443474 | -0.000752959 | 0.160284484 | 0.2548032 | 0.3517417 | 0.5756098 |
| Body Control Training | 170 | 0.2925621 | 0.1646976 | -0.016846243 | 0.178805614 | 0.2896731 | 0.3937484 | 0.6339017 |
| Body Control Training | 220 | 0.3089639 | 0.1723561 | -0.013855094 | 0.191066934 | 0.3054658 | 0.4167954 | 0.6564563 |
| Body Control Training | 280 | 0.3166227 | 0.1712947 | -0.003104257 | 0.197343146 | 0.3116763 | 0.4274599 | 0.6687635 |
| Body Control Training | 330 | 0.3129855 | 0.1659447 | 0.013684758 | 0.194403877 | 0.3080469 | 0.4207825 | 0.6656599 |
| Body Control Training | 390 | 0.2965975 | 0.1654541 | -0.002011494 | 0.178014619 | 0.290162 | 0.4035137 | 0.6427683 |
| Body Control Training | 440 | 0.2729214 | 0.1829979 | -0.068273595 | 0.149305583 | 0.2654449 | 0.3906998 | 0.6667157 |
| Body Control Training | 500 | 0.2324867 | 0.2370627 | -0.212661782 | 0.071938261 | 0.2264578 | 0.386556 | 0.7225949 |
| Resistance Training | 0 | 0.1686637 | 0.103736 | 0.024024695 | 0.091307291 | 0.1480913 | 0.2265325 | 0.4250405 |
| Resistance Training | 56 | 0.2651069 | 0.1387294 | 0.012928529 | 0.167933119 | 0.2577797 | 0.352319 | 0.5687162 |
| Resistance Training | 110 | 0.3354131 | 0.1875596 | -0.027593948 | 0.206157939 | 0.3341219 | 0.4653065 | 0.7087376 |
| Resistance Training | 170 | 0.3874 | 0.2270328 | -0.059632168 | 0.231033116 | 0.3892896 | 0.5427602 | 0.8314815 |
| Resistance Training | 220 | 0.4097107 | 0.2434504 | -0.076288758 | 0.242922416 | 0.4111555 | 0.5778322 | 0.8812419 |
| Resistance Training | 280 | 0.4112693 | 0.2436787 | -0.074554137 | 0.250069737 | 0.4128341 | 0.577345 | 0.8813346 |
| Resistance Training | 330 | 0.3915565 | 0.2308045 | -0.06334833 | 0.241564029 | 0.3889884 | 0.5453435 | 0.8434944 |
| Resistance Training | 390 | 0.3426869 | 0.210257 | -0.066523475 | 0.206920269 | 0.3348704 | 0.4783705 | 0.7671216 |
| Resistance Training | 440 | 0.2809505 | 0.2067298 | -0.118327428 | 0.140019061 | 0.2779825 | 0.4154273 | 0.7122557 |
| Resistance Training | 500 | 0.1816527 | 0.2484953 | -0.280011701 | 0.006903991 | 0.1807208 | 0.3509966 | 0.6818524 |

Supplementary Table 7. Summary of Evidence Quality for Exercise Dose on Motor Skill Improvement in Children and Adolescents with Cerebral Palsy (GRADE Assessment)

| **Outcome** | **Study design** | **Risk of bias** | **Inconsistency** | **Indirectness** | **Imprecision** | **Publication bias** | **Overall certainty of evidence** |
| --- | --- | --- | --- | --- | --- | --- | --- |
| Motor skill improvement (GMFM-66/88) | RCTs (n=20) | Moderate: Mean PEDro score 6.7 (SD=1.0); 85% ≥6 points; lack of blinding for participants/therapists in most studies | Mild: Overall I² indicated low-to-moderate heterogeneity; differences mainly across exercise subtypes | No: All studies targeted CP children/adolescents, with relevant interventions and outcomes | Moderate: Several trials with small sample sizes and wide CIs crossing the null | Borderline: Funnel plot showed acceptable symmetry but Egger’s test approached significance (p≈0.05) | **Moderate** |

Supplementary Figure 1. Network geometry of overall exercise at different dose levels.

Supplementary Figure 2. Predicted dose–response relationship of overall exercise from MBNMA with 95% credible intervals.

Supplementary Figure 3. Rank probability distributions of overall exercise doses based on MCMC simulations.

Supplementary Figure 4. Posterior mean distributions of overall exercise effects across dose levels.

Supplementary Figure 5. Observed treatment responses of overall exercise at different dose levels on the link scale.

Supplementary Figure 6. Network geometry of different exercise modalities across dose levels.

Notes: AEE, Aerobic Exercise; BCT, Body Control Training; RT, Resistance Training.

Supplementary Figure 7. Predicted dose–response relationship of different exercise modalities from MBNMA with 95% credible intervals.

Supplementary Figure 8. Rank probability distributions of different exercise modalities across dose levels based on MCMC simulations.

Supplementary Figure 9. Posterior mean distributions of different exercise modalities across dose levels.

Supplementary Figure 10. Observed treatment responses of different exercise modalities across dose levels on the link scale.

Supplementary Figure 11. Contour-enhanced funnel plot for publication bias assessment.

Supplementary Figure 12. Power-enhanced funnel plot for publication bias assessment.

Included studies:^[1-20]^

[1] DODD K J, TAYLOR N F, GRAHAM H K. A randomized clinical trial of strength training in young people with cerebral palsy [J]. Dev Med Child Neurol, 2003, 45(10): 652-7.<https://doi.org/10.1017/s0012162203001221>

[2] TSORLAKIS N, EVAGGELINOU C, GROUIOS G, et al. Effect of intensive neurodevelopmental treatment in gross motor function of children with cerebral palsy [J]. Dev Med Child Neurol, 2004, 46(11): 740-5.<https://doi.org/10.1017/s0012162204001276>

[3] LIAO H F, LIU Y C, LIU W Y, et al. Effectiveness of loaded sit-to-stand resistance exercise for children with mild spastic diplegia: a randomized clinical trial [J]. Arch Phys Med Rehabil, 2007, 88(1): 25-31.<https://doi.org/10.1016/j.apmr.2006.10.006>

[4] CHRISTIANSEN A S, LANGE C. Intermittent versus continuous physiotherapy in children with cerebral palsy [J]. Dev Med Child Neurol, 2008, 50(4): 290-3.<https://doi.org/10.1111/j.1469-8749.2008.02036.x>

[5] LEE J H, SUNG I Y, YOO J Y. Therapeutic effects of strengthening exercise on gait function of cerebral palsy [J]. Disabil Rehabil, 2008, 30(19): 1439-44.<https://doi.org/10.1080/09638280701618943>

[6] BAR-HAIM S, HARRIES N, NAMMOURAH I, et al. Effectiveness of motor learning coaching in children with cerebral palsy: a randomized controlled trial [J]. Clin Rehabil, 2010, 24(11): 1009-20.<https://doi.org/10.1177/0269215510371428>

[7] FOWLER E G, KNUTSON L M, DEMUTH S K, et al. Pediatric endurance and limb strengthening (PEDALS) for children with cerebral palsy using stationary cycling: a randomized controlled trial [J]. Phys Ther, 2010, 90(3): 367-81.<https://doi.org/10.2522/ptj.20080364>

[8] SCHOLTES V A, BECHER J G, COMUTH A, et al. Effectiveness of functional progressive resistance exercise strength training on muscle strength and mobility in children with cerebral palsy: a randomized controlled trial [J]. Dev Med Child Neurol, 2010, 52(6): e107-13.<https://doi.org/10.1111/j.1469-8749.2009.03604.x>

[9] JOHNSTON T E, WATSON K E, ROSS S A, et al. Effects of a supported speed treadmill training exercise program on impairment and function for children with cerebral palsy [J]. Dev Med Child Neurol, 2011, 53(8): 742-50.<https://doi.org/10.1111/j.1469-8749.2011.03990.x>

[10] CHRYSAGIS N, SKORDILIS E K, STAVROU N, et al. The effect of treadmill training on gross motor function and walking speed in ambulatory adolescents with cerebral palsy: a randomized controlled trial [J]. Am J Phys Med Rehabil, 2012, 91(9): 747-60.<https://doi.org/10.1097/PHM.0b013e3182643eba>

[11] DIMITRIJEVIĆ L, ALEKSANDROVIĆ M, MADIĆ D, et al. The effect of aquatic intervention on the gross motor function and aquatic skills in children with cerebral palsy [J]. J Hum Kinet, 2012, 32: 167-74.<https://doi.org/10.2478/v10078-012-0033-5>

[12] BRYANT E, POUNTNEY T, WILLIAMS H, et al. Can a six-week exercise intervention improve gross motor function for non-ambulant children with cerebral palsy? A pilot randomized controlled trial [J]. Clin Rehabil, 2013, 27(2): 150-9.<https://doi.org/10.1177/0269215512453061>

[13] CHEN C L, CHEN C Y, LIAW M Y, et al. Efficacy of home-based virtual cycling training on bone mineral density in ambulatory children with cerebral palsy [J]. Osteoporos Int, 2013, 24(4): 1399-406.<https://doi.org/10.1007/s00198-012-2137-0>

[14] GRECCO L A, ZANON N, SAMPAIO L M, et al. A comparison of treadmill training and overground walking in ambulant children with cerebral palsy: randomized controlled clinical trial [J]. Clin Rehabil, 2013, 27(8): 686-96.<https://doi.org/10.1177/0269215513476721>

[15] WANG T H, PENG Y C, CHEN Y L, et al. A home-based program using patterned sensory enhancement improves resistance exercise effects for children with cerebral palsy: a randomized controlled trial [J]. Neurorehabil Neural Repair, 2013, 27(8): 684-94.<https://doi.org/10.1177/1545968313491001>

[16] LABAF S, SHAMSODDINI A, HOLLISAZ M T, et al. Effects of Neurodevelopmental Therapy on Gross Motor Function in Children with Cerebral Palsy [J]. Iran J Child Neurol, 2015, 9(2): 36-41.

[17] CURTIS D J, WOOLLACOTT M, BENCKE J, et al. The functional effect of segmental trunk and head control training in moderate-to-severe cerebral palsy: A randomized controlled trial [J]. Dev Neurorehabil, 2018, 21(2): 91-100.<https://doi.org/10.1080/17518423.2016.1265603>

[18] CHO H J, LEE B H. Effect of Functional Progressive Resistance Exercise on Lower Extremity Structure, Muscle Tone, Dynamic Balance and Functional Ability in Children with Spastic Cerebral Palsy [J]. Children (Basel), 2020, 7(8).<https://doi.org/10.3390/children7080085>

[19] MOHAMED N, IBRAHIM M B, EL-AGAMY O A, et al. Effects of Core Stability Training on Balance, Standing, and Gait in Children with Mild Cerebral Palsy: A Randomized Controlled Trial [J]. Healthcare, 2025, 13(11).<https://doi.org/10.3390/healthcare13111296>

[20] SAKZEWSKI L, BLEYENHEUFT Y, NOVAK I, et al. A Multisite Randomized Controlled Trial of Hand Arm Bimanual Intensive Training Including Lower Extremity for Children with Bilateral Cerebral Palsy [J]. Journal of Pediatrics, 2025, 284.<https://doi.org/10.1016/j.jpeds.2025.114666>
