# Supplementary material for: Overall and modality-specific exercise doses for motor skill improvement in cerebral palsy: a systematic review and Bayesian network dose-response meta-analysis
Source: PeerJ. 2026 Apr 8;14:e21035. doi: 10.7717/peerj.21035 (PMC13069938; doi:10.7717/peerj.21035)
Supplement: Supplemental Information 15 [file peerj-14-21035-s015.docx]

**Intended Audience**

This systematic review and network meta-analysis is intended for the following audiences:

**Clinical and Rehabilitation Professionals**
Including rehabilitation physicians, physical therapists, occupational therapists, and other practitioners involved in designing exercise-based interventions for children with cerebral palsy. The findings provide evidence-based guidance for determining appropriate exercise dose and structuring individualized rehabilitation programs.

**Researchers in Exercise Science and Public Health**
Including scientists focusing on exercise dosing, intervention efficacy, neurophysiological mechanisms, pediatric motor disorders, and methodological development in dose–response modeling. The standardized dose metric (METs·min/week) and modeled dose–response curves support cross-study comparison and further research advancement.

**Policy-makers and Guideline Developers**
Including individuals involved in drafting clinical practice guidelines, rehabilitation pathways, and public health recommendations for pediatric motor rehabilitation. This study provides quantitative evidence to support dose-oriented prescribing and the development of more precise, evidence-based clinical standards.
